# Mapping protein interactions in the active TOM-TIM23 supercomplex

**DOI:** 10.1038/s41467-021-26016-1

**Published:** 2021-09-29

**Authors:** Ridhima Gomkale, Andreas Linden, Piotr Neumann, Alexander Benjamin Schendzielorz, Stefan Stoldt, Olexandr Dybkov, Markus Kilisch, Christian Schulz, Luis Daniel Cruz-Zaragoza, Blanche Schwappach, Ralf Ficner, Stefan Jakobs, Henning Urlaub, Peter Rehling

**Affiliations:** 1 Department of Cellular Biochemistry, University Medical Center Göttingen, Göttingen, Germany; 2 Department of Clinical Chemistry, University Medical Center Göttingen, Göttingen, Germany; 3 Bioanalytical Mass Spectrometry Group, Max Planck Institute for Biophysical Chemistry, Am Fassberg 11, Göttingen, Germany; 4 Department for Molecular Structural Biology, Georg-August-Universität Göttingen, Göttingen, Germany; 5 Department of NanoBiophotonics, Max Planck Institute for Biophysical Chemistry, Göttingen, Germany; 6 Clinic of Neurology, University Medical Center Göttingen, Göttingen, Germany; 7 Cellular Biochemistry, Max Planck Institute for Biophysical Chemistry, Am Fassberg 11, Göttingen, Germany; 8 Department of Molecular Biology, University Medical Center Göttingen, Göttingen, Germany; 9 Cluster of Excellence “Multiscale Bioimaging: from Molecular Machines to Networks of Excitable Cells” (MBExC), University of Göttingen, Göttingen, Germany; 10 Max Planck Institute for Biophysical Chemistry, Göttingen, Germany

**Keywords:** Membrane proteins, Mitochondrial proteins, Mitochondria, Protein translocation, Molecular modelling

## Abstract

Nuclear-encoded mitochondrial proteins destined for the matrix have to be transported across two membranes. The TOM and TIM23 complexes facilitate the transport of precursor proteins with N-terminal targeting signals into the matrix. During transport, precursors are recognized by the TIM23 complex in the inner membrane for handover from the TOM complex. However, we have little knowledge on the organization of the TOM-TIM23 transition zone and on how precursor transfer between the translocases occurs. Here, we have designed a precursor protein that is stalled during matrix transport in a TOM-TIM23-spanning manner and enables purification of the translocation intermediate. Combining chemical cross-linking with mass spectrometric analyses and structural modeling allows us to map the molecular environment of the intermembrane space interface of TOM and TIM23 as well as the import motor interactions with amino acid resolution. Our analyses provide a framework for understanding presequence handover and translocation during matrix protein transport.

## Abstract (web summary)

The TOM and TIM23 complexes facilitate the transport of nuclear-encoded proteins into the mitochondrial matrix. Here, the authors use a stalled client protein to purify the translocation supercomplex and gain insight into the TOM-TIM23 interface and the mechanism of protein handover from the TOM to the TIM23 complex.

## Introduction

Multiple import pathways facilitate the transport of mitochondrial proteins from the cytosol into the various mitochondrial compartments. The TOM complex (translocase of the outer mitochondrial membrane) serves as the general entry gate for mitochondrial proteins^1–3^. One of the predominant mitochondrial protein import pathways, is the so-called presequence pathway, which is dedicated to the transport of precursor proteins with N-terminal, amphipathic α helical targeting signals, presequence, across the inner membrane. Along the transport pathway across the outer and inner mitochondrial membranes, the presequence is sequentially recognized by several receptors thereby providing translocation specificity and velocity^4–6^.

Precursors are recognized on the outer surface of mitochondria by cytosol-exposed receptors Tom20 and Tom22 that are functionally supported by other presequence-binding TOM subunits Tom5, Tom70, and the pore-forming Tom40^7–11^. At the exit of the Tom40 channel, the intermembrane space (IMS) domain of Tom22 serves as a *trans*-binding site for the presequence^12–14^. Recent structural and biochemical analyses of the *Saccharomyces cerevisiae* TOM complex provide us with a blueprint on how precursors are transported through the TOM complex^2,3,15^. However, the subsequent steps of transport are not well understood.

For inner membrane translocation, precursor proteins need to be handed over from the outer membrane TOM complex to the TIM23 complexes in the inner membrane. Once precursor translocation across the inner membrane is achieved, a transient translocation intermediate of a TOM-TIM23-spanning precursor is formed demonstrating that the translocation processes across the two membranes occur in a synchronized manner^16–19^. Accordingly, for the handover of the precursor from TOM to the TIM23 complex, both complexes need to be in proximity to each other. A complex set of protein interactions among IMS-domains of Tom22, Tim50, Tim23, and Tim21 and with the presequence of the incoming precursor are thought to facilitate precursor transfer across the IMS to the TIM23 complex. However, a molecular understanding of these processes in the context of the translocation machineries is still missing. For precursor recognition at the inner membrane, the Tim50^IMS^-domain acts as the primary presequence receptor. In addition to Tom22^IMS^, Tim50^IMS^, and Tim23^IMS^ interact with presequences in the intermembrane space^20–25^. Tim21^IMS^ promotes dissociation of the precursor from Tom22^IMS^^26,27^.

For inner membrane translocation, the Tim23 channel is regulated in a precursor-dependent manner: Tim23^IMS^ facilitates dimerization of the channel-forming Tim23 protein in a presequence and membrane potential-dependent manner^28–30^. In the presence of presequences, the dimer dissociates, leading to activation of the channel^28,29,31^. The membrane potential subsequently drives the positively charged presequence across the inner membrane. For full translocation of the precursor across the membrane, Tim44 and mtHsp70 of the PAM complex engage the polypeptide chain at the exit of the Tim23 channel. During transport of the polypeptide, the ATPase activity of Hsp70 has to be regulated by the Pam18 (Tim14) J-protein and its regulator Pam16 (Tim16)^32–38^.

Despite the available biochemical data on individual protein interactions and receptor functions, the complex mechanism of protein transfer, along with the interplay of interactions between the TOM, TIM23, and PAM complex subunits during import of a protein are not understood. Moreover, we lack spatial information on the organization of the translocase components that enable precursor transfer between outer and inner membranes.

In this work, we utilize a precursor protein with a tightly folded C-terminal blocking moiety to generate and stabilize the TOM-TIM23 translocation intermediate both in vivo and in vitro. Utilizing a cross-linking-based mass spectrometry approach, we define interactions of translocase constituents in the presence and absence of accumulated precursor with amino acid resolution. Based on molecular modeling approaches of protein interactions, we propose a model of the TOM-TIM23 transition zone and provide insight into precursor transport from the outer membrane TOM to the inner membrane TIM23 complex and on interactions in the mitochondrial import motor.

## Results and discussion

### A supercomplex-stabilizing precursor stalled in mitochondrial import

**Fig. 1:**
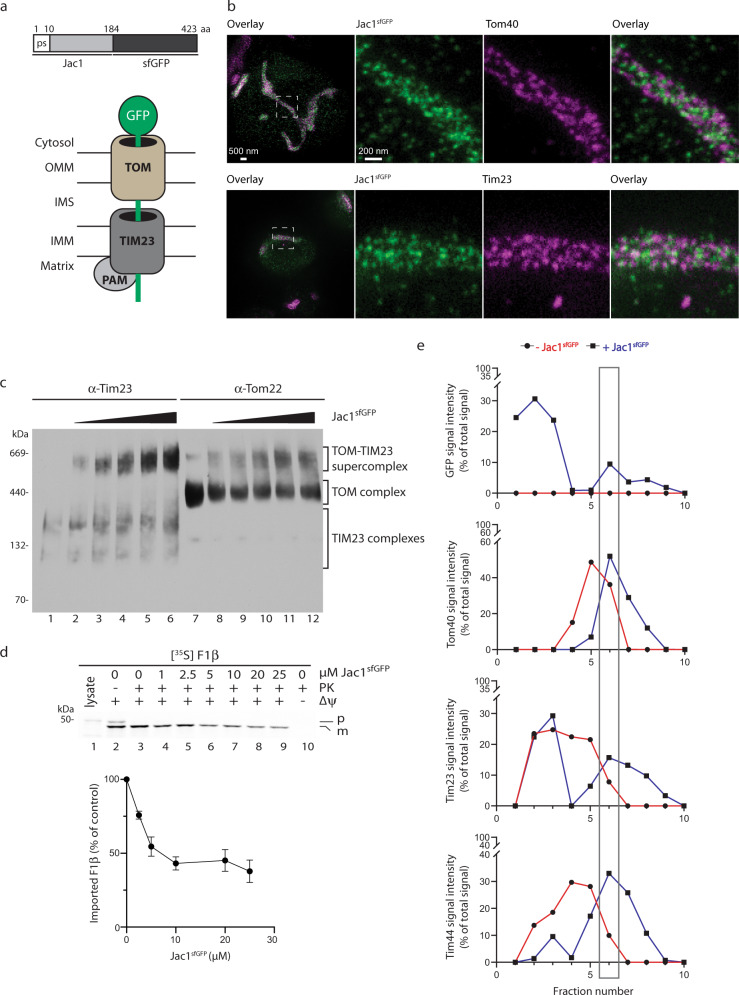
Jac1^sfGFP^ localizes to mitochondria and accumulates in a TOM-TIM23 supercomplex. **a** Design of Jac1^sfGFP^ supercomplex forming protein and its schematic representation within the TOM-TIM23 supercomplex. **b** STED super-resolution microscopy of yeast cells expressing Jac1^sfGFP^. Cells were labeled with antibodies against GFP (green) and Tom40 (magenta) or Tim23 (magenta). Representative images from three biological replicates are depicted. **c** Increasing amounts of purified Jac1^sfGFP^ were imported into wild-type mitochondria. Mitochondria were solubilized and supercomplex formation was monitored by BN-PAGE followed by immunoblotting using indicated antibodies. Four independent experiments were carried out. **d** Indicated amounts of purified Jac1^sfGFP^ were imported for 30 min into 50 µg mitochondria. Mitochondria were briefly washed and [^35^S]-labeled F1β was imported for 15 min. The reaction was stopped by the addition of AVO followed by PK (Proteinase K) treatment. Samples were analyzed by SDS-PAGE and digital autoradiography. The imported precursor was quantified as percent of the 0 µM protein sample with PK treatment (100% control) (lane 3). Results are presented as mean ± SEM, *n* = 4. **e** After import, solubilized mitochondria with or without imported Jac1^sfGFP^ were subjected to glycerol gradient centrifugation. Fractions were analyzed by SDS-PAGE and immunoblotting. The signal intensity of supercomplex subunits were quantified and normalized as % of the total signal. ps: presequence, aa: amino acid, sfGFP: superfolder GFP, TOM: translocase of the outer mitochondrial membrane, TIM23: presequence translocase of the inner mitochondrial membrane, PAM: presequence translocase-associated motor complex, OMM: outer mitochondrial membrane, IMS: intermembrane space, IMM: inner mitochondrial membrane, p: precursor, m: mature (processed) form, AVO: a mixture of Antimycin A, Valinomycin Oligomycin.

Transport of presequence-containing mitochondrial proteins requires cargo handover from the outer membrane TOM to the inner membrane TIM23 complex. Upon inner membrane translocation of the precursor a translocation intermediate spanning both TOM and TIM23 complexes is established by the precursor^39^. At the analytical level, the 20 nm long TOM-TIM23 supercomplex could be stabilized and monitored upon import of a mitochondrial precursor with a C-terminally fused folded domain that cannot pass the TOM complex^16–18,40^. Here we designed a precursor protein consisting of the N-terminus of the mitochondrial presequence-containing Jac1 fused to superfolder GFP (sfGFP) to stabilize translocases in a supercomplex translocation intermediate (Fig. 1a). Upon expression in *Saccharomyces cerevisiae* cells, co-localization of the Jac1^sfGFP^ precursor with mitochondria was observed by fluorescence microscopy (Supplementary Fig. 1a, b). Furthermore, Jac1^sfGFP^ and translocase subunits displayed the expected high degree of proximity in STED super-resolution images (Fig. 1b). To define the supercomplex-forming capacity of Jac1^sfGFP^, we purified the fusion protein and performed in vitro import into mitochondria. Analysis of the translocase complexes TOM and TIM23 by Blue Native (BN)-PAGE revealed precursor-dependent formation of a TOM-TIM23 supercomplex that increased in abundance with the amounts of added Jac1^sfGFP^ (Fig. 1c). In agreement with the formation of a TOM-TIM23 supercomplex occupied by Jac1^sfGFP^, accumulation of increasing amounts of Jac1^sfGFP^ in mitochondria led to saturation of presequence import sites, reflected by reduced import capacity for a [^35^S]-labeled mitochondrial matrix protein F_1_β (Atp2) (Fig. 1d).

When solubilized mitochondrial protein complexes were analyzed by glycerol gradient centrifugation, TOM and TIM23 complex subunits displayed a Jac1^sfGFP^-dependent shift in their running pattern towards higher molecular weight fractions (Supplementary Fig. 1c). Signal intensity analyses revealed the TOM-TIM23 supercomplex to be predominantly migrating in fraction 6 (Fig. 1e). We concluded that, the Jac1^sfGFP^ fusion protein localized to mitochondria in vivo and in vitro and led to the formation of a precursor saturated translocase supercomplex consisting of TOM and TIM23 complexes.

### Purification of the TOM-TIM23 supercomplex

**Fig. 2:**
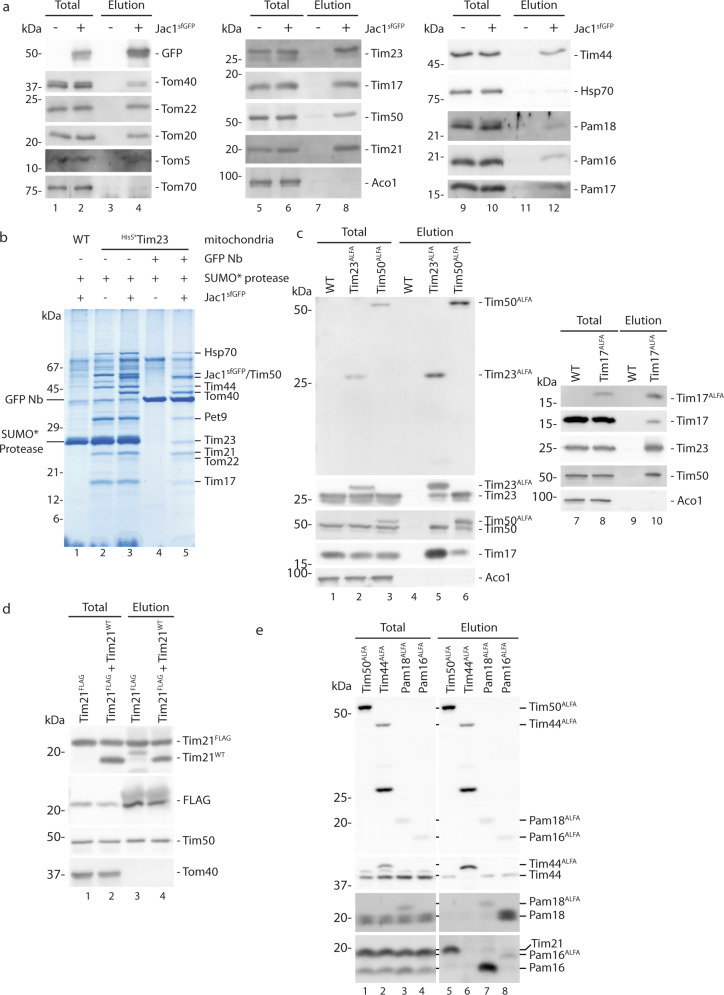
Purification of TOM-TIM23 supercomplex. **a** Jac1^sfGFP^ was imported into wild-type mitochondria and the TOM-TIM23 supercomplex isolated using a GFP nanobody. Samples were analyzed by SDS-PAGE and western blotting. Representative image from *n* > 3 biological replicates. **b** Preparative isolation of the TIM23 complex from ^HisS*^Tim23 (^His^SUMOstar-Tim23) mitochondria with or without accumulated Jac1^sfGFP^. After SUMO* protease-mediated elution, the TOM-TIM23 supercomplex was specifically purified with GFP nanobody (Nb). Samples were analyzed by SDS-PAGE, colloidal coomassie staining, and mass spectrometry. The experiment was repeated independently for *n* > 3. Plasmids containing ALFA- tagged **c** Tim23, Tim17, and Tim50 and **e** Tim50, Tim44, Pam16, and Pam18 were transformed into WT yeast. Mitochondria isolated from these cells were solubilized and subjected to ALFA immunoprecipitation. Total and elution fractions were analyzed by SDS-PAGE and immunoblotting. ALFA-tagged proteins (upper panel) were detected using an anti-ALFA Nanobody conjugated with HRP. **d** Mitochondria were isolated from Tim21^FLAG^ expressing cells transformed with empty plasmid or plasmid encoding untagged Tim21 (TIM21^WT^). Following FLAG immunoisolation, samples were analyzed by SDS-PAGE and western blotting. For **c**−**e**, experiments were repeated for *n* = 3.

To define the supercomplex biochemically, we solubilized wild-type (WT) mitochondria following the import of Jac1^sfGFP^ and immunoisolated the translocation intermediate using a GFP Nanobody (Nb) coupled to sepharose. As expected, together with the GFP-containing precursor, components of the TOM (Tom40, Tom22, Tom20, Tom5), TIM23 (Tim23, Tim17, Tim50, Tim21) and PAM (Tim44, Hsp70, Pam18, Pam16) complexes were specifically isolated (Fig. 2a). For preparative TOM-TIM23 supercomplex purification, we devised a two-step approach. First, a yeast strain expressing ^His^SUMOstar-Tim23 (^HisS*^Tim23) was generated allowing for native TIM23 complex isolation via His-tagged Tim23 followed by SUMOstar protease cleavage^41^. As the second purification step, GFP-Nb affinity purification was applied. The TIM23 complex was isolated in the presence and absence of imported Jac1^sfGFP^. Upon ^HisS*^Tim23 purification, a similar pattern of purified proteins was apparent in both conditions (Fig. 2b, lanes 2 and 3), except for additional bands of Jac1^sfGFP^ and Tom40 in the sample with accumulated Jac1^sfGFP^ (lane 3). We then applied these eluted samples to a GFP Nb column, to specifically isolate supercomplex components. The purified fraction was specifically enriched in components of the TOM, TIM23, and PAM complexes (Fig. 2b, lane 5), as confirmed by immunodetection and mass spectrometry. Thus, as has been previously established, both the Tim21-containing motor free and the Tim21-free PAM-containing forms of the supercomplex were isolated^42^. Accordingly, the TOM-TIM23 supercomplex could be natively purified from mitochondria after import of Jac1^sfGFP^ in preparatory scale. Hence, this strategy represents a means to biochemically purify and define supercomplex organization.

Next, we addressed the oligomerization state of selected Tim and Pam subunits in the translocase. To this end, we utilized yeast strains, which expressed a wild-type copy of the protein of interest and a tagged variant. ALFA-tagged Tim23, Tim17, Tim50, Tim44, Pam16, and Pam18 were expressed in yeast along with their corresponding wild-type variant. Solubilized mitochondria were subjected to immunoisolation using a nanobody directed against the ALFA tag. The TIM23 complex subunits Tim23^ALFA^, Tim17^ALFA^, and Tim50^ALFA^ clearly co-isolated their corresponding wild-type counterpart (Fig. 2c). Hence, more than one copy of Tim23, Tim17, and Tim50 were present in the TIM23 complex. For Tim21, a FLAG-tagged variant was used for immunoisolation. Both, wild-type Tim21 and Tim21^FLAG^ were present in the purified fraction, indicating that more than one copy of Tim21 is present in the TIM23 complex (Fig. 2d). In case of the motor complex subunits Tim44^ALFA^, Pam18^ALFA^, and Pam16^ALFA^, none of the tagged proteins copurified its wild-type counterpart in significant amounts, indicating that Tim44, Pam16, and Pam18 are most likely present as a single copy at the translocase (Fig. 2e). Taken together, TIM23 complex subunits are mostly present in multiple copies in the translocase while motor subunits predominantly appear to be present in a single copy.

### Dissecting translocase organization by chemical cross-linking

To define protein organization and dynamics during protein import, we combined complex isolation with chemical cross-linking. To this end, the TIM23 complex was isolated following the import of Jac1^sfGFP^. The purified complex was subjected to chemical cross-linking using amino-reactive homobifunctional long spacer cross-linkers such as DSS (11.4 Å spacer) and DSSO (10.1 Å spacer) and heterobifunctional short spacer length cross-linkers, e.g., SDA (3.9 Å spacer) and EDC (0 Å spacer), to cover maximal protein cross-links at various spacer distance constraints. In western blot analyses, we observed cross-linked adducts of Tom20, Tom22, Tom40, Tim21, Tim23, and Tim44 upon DSS, DSSO, and EDC treatment (Supplementary Fig. 2a). Based on this, we subjected cross-linked samples obtained from DSS-, SDA-, and EDC-mediated cross-linking to mass spectrometric analyses to define protein interaction sites with amino acid resolution (Supplementary Data 1). To corroborate the cross-link data obtained for the purified protein complexes, we carried out two complementary approaches. First, we performed *in organello* cross-linking with DSS and EDC, and second, we performed *in organello* cross-linking with DSS followed by TIM23 complex isolation (Supplementary Data 1).

### Interactions of the TIM23 complex

**Fig. 3:**
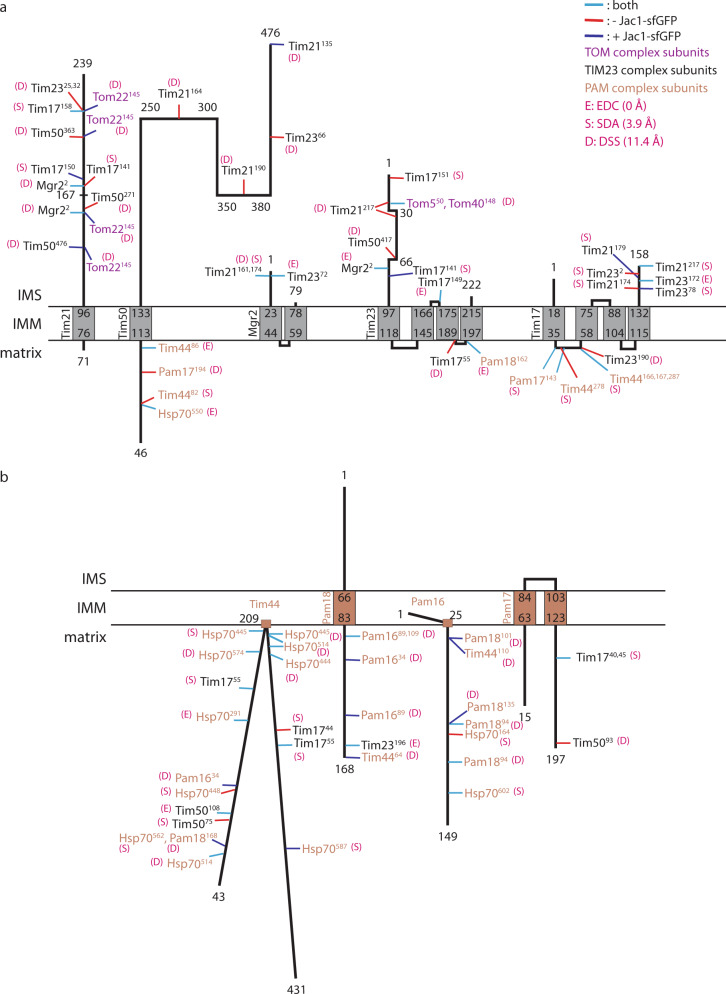
Analyses of cross-links in the TOM-TIM23 supercomplex. Schematic representation of inter-protein cross-links identified for **a** TIM23 complex subunits and **b** PAM complex subunits by LC-MS/MS analysis following cross-linking of the isolated complex with DSS (11.4 Å spacer), EDC (0 Å spacer), and SDA (3.9 Å spacer). The cross-links can be found in Supplementary Data 1, approach 1, and Supplementary Data 2. Numbers indicate amino acid residue. IMS: intermembrane space, IMM: inner mitochondrial membrane.

The cross-linking data allowed us to define interaction sites between TOM, TIM23, and PAM (presequence translocase-associated import motor) complex constituents (Fig. 3a, b). The IMS domain of the Tim50 receptor displayed multiple interactions with the IMS-localized N-terminus of the Tim23 channel and Tim21^IMS^ (Fig. 3a). The interaction of Tim50^IMS^ could be mapped to the predicted coiled-coil domain of Tim23^IMS^, which is in agreement with a role of Tim50 in Tim23 oligomerization^28,30,39,40^. Interestingly, the Tim50/Tim23 cross-link was only detected in the absence of a precursor, suggesting that a conformational change in Tim50^IMS^ alters its relative position to Tim23 in the open state of the translocation channel. The Tim23 channel showed multiple interactions with Tim17. Cross-links between Tim17 and Tim23^IMS^ were preferentially obtained with short distance cross-linkers EDC and SDA and the matrix cross-link with the longer distance cross-linker DSS (Fig. 3a and Supplementary Data 1). Moreover, the C-terminus of Tim17 appeared to be positioned in proximity to Tim21^IMS^ and also cross-linked to several regions along the polypeptide chain of Tim23 (Fig. 3a).

Interestingly, we observed cross-links between Tim17 and the extreme N-terminus of Tim23 (aa 2). The topology of the N-terminus of Tim23 has remained controversial in the field as it has been proposed to localize to the cytosolic face of the outer membrane^43,44^. In our opinion, the presence of an interaction between the N-terminus of Tim23 and Tim17 clearly favors the concept of a location of the Tim23 N-terminus in the IMS (see also below). Furthermore, using Tim23 cross-linking information from isolated complex and *in organello* cross-linking approaches, we obtained multiple intra-protein cross-links throughout the putative Tim23^IMS^, and inter-protein cross-links with IMS-exposed domains of other translocase subunits (Supplementary Fig. 3a). To further support an intermembrane space localization of the TIM23^IMS^, we generated single cysteine Tim23 substitutions at various positions within the first 60 amino acids, in a *TIM23* deletion background. These mutants complemented the lethal *tim23Δ* growth phenotype and purified mitochondria displayed wild-type-like protein levels (Supplementary Fig. 3b). Mitochondria and mitoplasts from these mutants were subjected to a cysteine modification assay using maleimide activated streptavidin, that cannot pass the intact outer membrane. For all mutants, Tim23-specific modified bands could be observed only in mitoplasts, indicating the localization of these residues in the IMS (Supplementary Fig. 3c). Together, these results support the idea that the N-terminus of Tim23 is localized within the intermembrane space.

The extreme N-terminus of the Mgr2 gate keeper protein displays cross-links to Tim21^IMS^ and Tim23^IMS^. Considering that the Tim23 channel dimerizes through the N-terminal domain, it is tempting to speculate that the lateral gate for membrane insertion of precursors, which is controlled by Mgr2, is positioned at the N-terminal portion of Tim23. In addition to the cross-link of Mgr2 to Tim23^IMS^, we also detected cross-links to Tim21^IMS^, both of which were found in the presence and absence of an accumulated precursor. The IMS domain of Tim21 appears to represent a hot spot of interactions. Within the TIM23 complex, Tim21^IMS^ reveals cross-links to Tim50^IMS^, Tim17 C-terminus, and Mgr2. While cross-links between Tim21^IMS^ aa 161,164, and 190 and Tim50^IMS^ were detected only in the absence of a precursor, Tim21^IMS^ aa 135 cross-linked to the extreme C-terminus of the Tim50^IMS^ was only detected in the presence of a precursor, suggesting that the C-terminal presequence-binding domain of Tim50^22^ folds towards Tim21 once the precursor has been passed to the channel.

The mitochondrial import motor associates to the TIM23 complex at the matrix side of the inner membrane to drive matrix transport of precursors. The cross-linking analyses identified multiple interaction sites between the import motor and the membrane module constituents of the TIM23 complex (Fig. 3b). At the matrix side of the inner membrane, Tim50^matrix^ contacts the N-terminal half of the motor constituent Tim44 and mtHsp70, as observed using short spacer cross-linkers. A role of Tim50 in the import of matrix proteins has been observed previously and a recent study suggested a function for Tim50 in PAM recruitment to the translocase^17,18,45–48^. The observed direct interaction between Tim44 and Tim50 provides a molecular explanation for this Tim50 function. In addition, the first loop of Tim17 is cross-linked to Pam17 C-terminal matrix segment, and also to Tim44, the latter being in agreement with the previous analyses^49^. The PAM complex cross-links are further discussed in a subsequent section. In summary, these analyses allow us to propose domain positions within the presequence translocase and the PAM complex and indicate that upon precursor translocation subunits undergo dynamic positional rearrangements relative to each other.

### Defining precursor positioning in the supercomplex and the TOM–TIM23 junction

Upon exit from the TOM complex, the precursor’s presequence becomes exposed to the intermembrane space and the IMS-exposed domains of supercomplex components engage with the amphipathic helix to facilitate the handover of the precursor from TOM to TIM23. For this, the two complexes need to be in close proximity to each other. We, therefore, aimed to utilize the available data to obtain a molecular model of the TOM-Tim23 interface during precursor transfer.

**Fig. 4:**
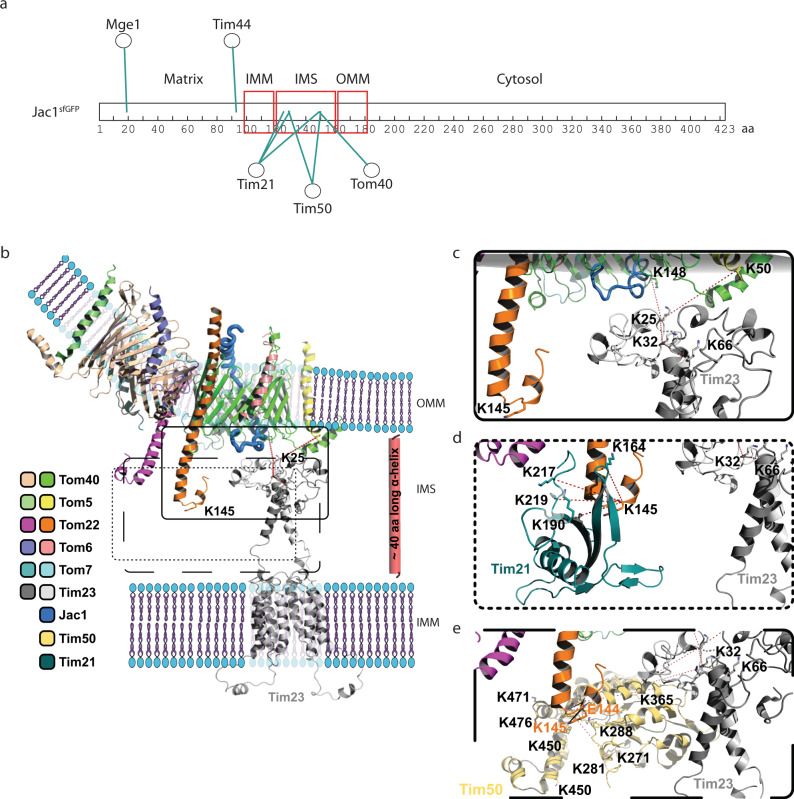
N-terminus of Tim23 is localized in proximity to the TOM complex. **a** Mapping of cross-links obtained between Jac1^sfGFP^ and various supercomplex subunits, indicating its relative position within the supercomplex. **b** Structural model representation of Tim23 relative to the TOM complex, indicating cross-links (red dashed line) between Tim23 (K25, gray), Tom40 (K148, green), and Tom5 (K50, yellow) in the intermembrane space (IMS). Red helix represents the region of Jac1^sfGFP^ predicted to be localized in the IMS based on (**a**). The model utilizes structural information from available TOM complex: 6JNF and Tim23: 7CLV structures. The color code indicates individual subunits. **c** Close-up view of the Tim23-Tom40 and Tim23-Tom5 cross-links. **d** Illustration showing Tim21-Tom22 cross-links using the structure of the IMS domains of Tim21 (cyan, PDB id: 2CIU). **e** Interaction between Tim50-Tim23 and Tim50-Tom22 based on cross-links. Tim50 (mustard) is indicated as a combination of the known structure of Tim50^CORE^ (PDB id: 3QLE) and an ab initio predicted model for the C-terminal domain. OMM: outer mitochondrial membrane, IMS: intermembrane space, IMM: inner mitochondrial membrane.

The obtained cross-linking data defined the position of the accumulated Jac1^sfGFP^ precursor in the translocases. The N-terminal residues 19 and 93 cross-linked to the matrix localized Mge1 and Tim44 respectively. Additionally, cross-linked peptides of Jac1^sfGFP^ (residues 125−150) were obtained with the IMS-exposed domains of Tim21 and Tim50, and the Tom40 channel. Accordingly, this region represents the IMS-exposed portion of the arrested precursor (Fig. 4a). Taking into consideration, that approximately 23 amino acids helical segment with a length of 35 Å suffices to span a lipid bilayer, we estimated based on cross-linking analysis of Jac1^sfGFP^ that a 40 amino acid segment in most likely alpha-helical conformation could be used as a molecular ruler spanning the intermembrane space (Fig. 4b).

We used the available TOM complex structure (PDB id: 6JNF)^2^ and completed undefined Tom40 domains by using the Rosetta tools designed for atomic model refinement and rebuilding against low-resolution cryo-EM maps. The missing IMS domain of Tom22 was built by using ab initio folding in Rosetta (Supplementary Fig. 4a, b). For positioning of the TIM23 complex, we employed available NMR-based structural information on Tim23 (PDB id: 7CLV)^50^, and remodeled the flexible 60 amino acid long N-terminal tail using cross-link guided molecular modeling. The available X-ray structure of Jac1 could be used to model the N-terminal portion of the precursor (PDB id: 3UO3)^51^. The calculated ideal 40 amino acid alpha-helix positioned between TOM and TIM23 together with the cross-links between the N-terminus of Tim23^IMS^ (K25) and Tom5 (K50) and Tom40 (K148) allowed us to position the Tim23^IMS^ at the TOM complex (Fig. 4b, c).

The model of Tim21 was prepared based on crystal structure 2CIU^27^. The atomic model of the N-terminal fragment of Tim50^IMS^ domain (residues 171−361) was obtained from the PDB (PDB id: 3QLE)^24^. The missing C-terminal fragment of Tim50 (residues 365−488) was modeled using cross-link guided molecular modeling utilizing Rosetta ab initio folding^52^. Three sets of at least 150,000 decoys were generated employing different amounts of cross-link derived spatial restraints. Low Rosetta score models were further subjected to clustering using MaxCluster. The best model of C-terminal fragment of Tim50 was selected based on the Rosetta score.

**Fig. 5:**
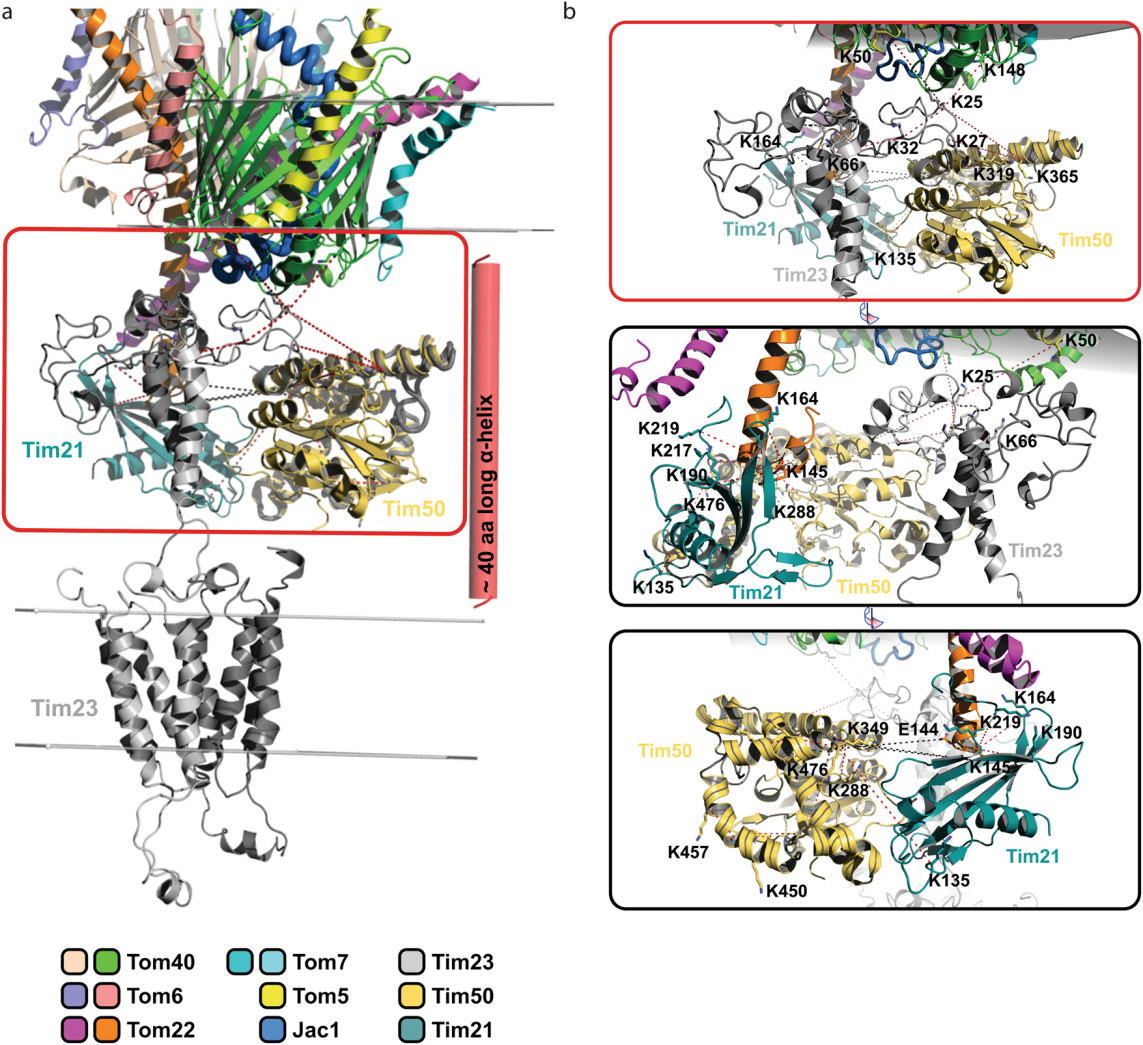
Overview of inter-protein interactions in the IMS. **a** Schematic representation of the overall model of the TOM-TIM23 supercomplex in the presence of Jac1^sfGFP^ (blue ribbon), based on cross-links. **b** Zoomed inset from (**a**) in three different orientations, indicating cross-links between IMS domains of Tom22 (orange), Tim21 (cyan), Tim23 (gray), and Tim50 (mustard).

Previously, negatively charged residues on Tom22^IMS^ have been shown to interact with positively charged residues on the Tim21^IMS^ surface^27^. Here, we obtained cross-links between K145 of Tom22 with multiple residues of Tim21^IMS^ (Supplementary Data 1) in the presence of Jac1^sfGFP^. These interaction sites locate to the outer surface of the β-sheet and the β-hairpin (Fig. 4d). This data was corroborated by western blotting analyses of cross-links between Tom22 and Tim21. In the absence of Tim21, the purified complex did not display the higher molecular weight cross-link adducts of Tom22 (Supplementary Fig. 2b). For incorporating the IMS-domain of Tim50 into the model, we utilized the cross-links with Tim23 and Tom22 as positional constraints (Fig. 4e). EDC cross-linking of whole mitochondria revealed interaction of Tom22 E144 and K145 with Tim50^IMS^. Additionally, multiple cross-links were obtained between Tim23^IMS^ and Tim50^IMS^ (Supplementary Data 1). Interestingly, Tim50^IMS^ cross-links occurred at different faces of Tim21 in the absence and presence of the precursor (Supplementary Data 1), indicating a positional or conformational rearrangement upon protein import. This finding is in line with the observation that presequences trigger dissociation of Tim21 from Tim50^25^. Together, Tim21, Tim23, and Tim50 facilitate interactions with the TOM complex during precursor transport and position the receptor Tim50^IMS^ and Tim23^IMS^ for recognition of a precursor (Fig. 5a). The proximity between various TOM and TIM components in the IMS would therefore promote its efficient handover (Fig. 5b).

**Fig. 6:**
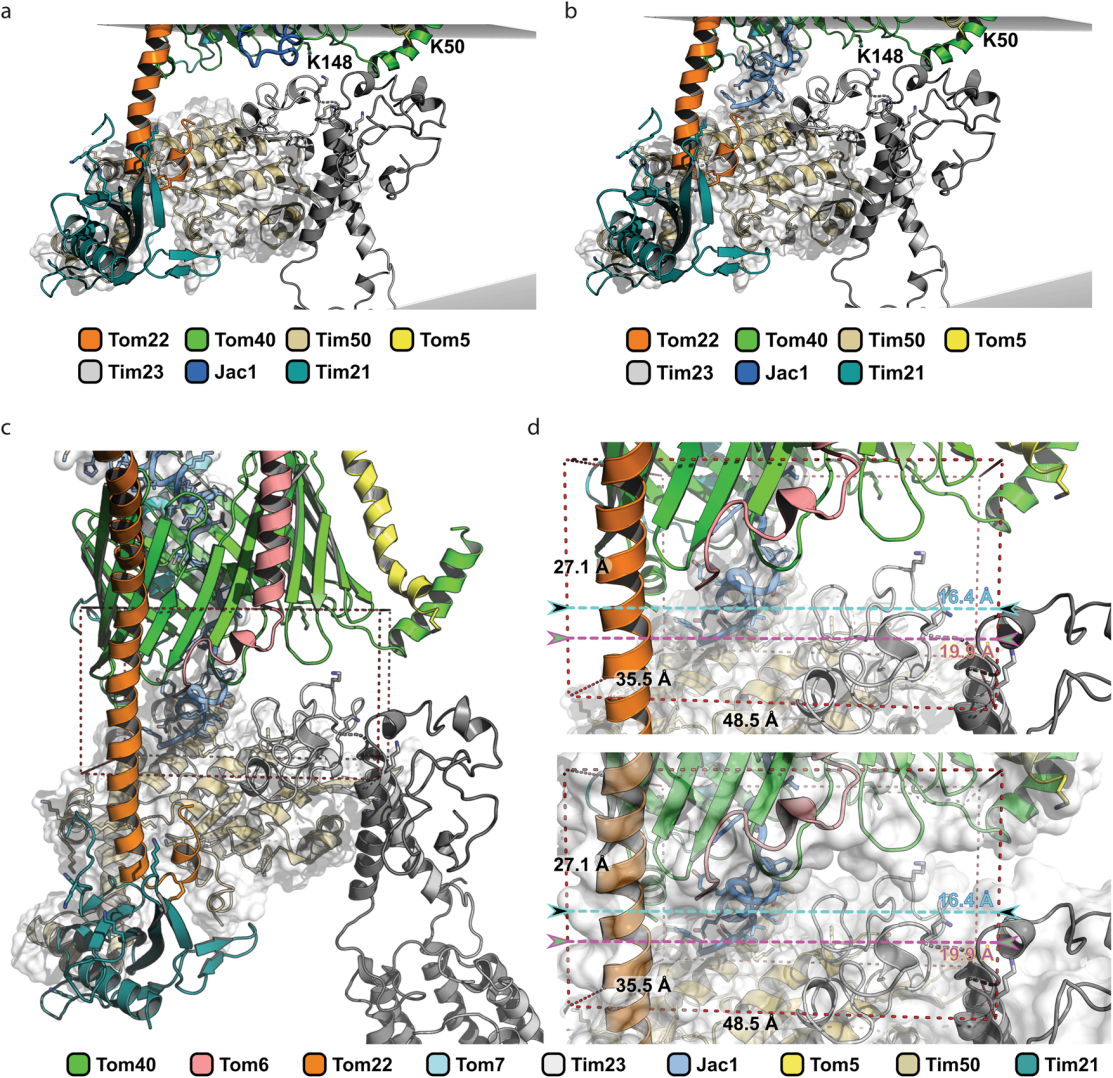
Model of presequence handover in the IMS. Surrounding components of Tom40 in the context of exiting Jac1 presequence towards **a** Tom22 and **b** Tim50, facilitating its stepwise transfer from the TOM to the TIM23 complex. **c** Dimensions of a cuboid encapsulating the spatial volume available in the IMS for a presequence upon its exit from the TOM pore. Numbers indicate distances in Å. **d** Close-up view of the cuboid in (**c**). The color code indicates individual subunits.

Based on our cross-link-based atomic model, we estimated the possible volume available for a presequence exiting the Tom40 pore into the intermembrane space. This volume was determined as approx. ranging between 28,237 Å^3^ or 2.82 × 10^−23^ L and 46,659 Å^3^ or 4.66 × 10^−23^ L (Fig. 6c, d). Accordingly, the concentration of a presequence in that given IMS space could be up to 35.6−58.8 mM (Fig. 6c, d). Interestingly, the relatively low presequence affinity of the IMS-exposed receptors determined in solution was in the range of 0.5 mM in case of Tim23^IMS^ and 4−45 µM Tim50^IMS^^20,21,25^. Accordingly, the association of the receptors with the presequence is favored in the TOM-TIM23 restricted space. Moreover, the proximity of the receptor domains at the *trans* side of the TOM complex would allow direct handover of the precursor between the receptor domains. To this end, the organization of the TOM-TIM23 interaction zone points towards a presequence affinity trap model, wherein a high association and dissociation rate of a presequence with IMS-exposed translocase subunits could facilitate efficient handover and transport from the TOM to the TIM23 complex, following which it encounters the PAM complex in the matrix.

### Interaction of Tim44 with presequences

In agreement with Tim44´s role as a scaffold protein, our analysis confirmed multiple interactions between Tim44 and motor components including Pam16, Pam18, and mtHsp70 (Fig. 3b). Cross-links of Tim44 were solely obtained with the C-terminal substrate-binding domain of mtHsp70. In case of the matrix-exposed Pam16 and Pam18, these proteins were only cross-linked to the N-terminus of Tim44 and in a precursor-dependent manner. Moreover, Pam18 and Pam16 displayed several cross-links with each other. In this case, we observed precursor-dependent and -independent cross-links indicative of conformational dynamics of the heterodimer. In addition, the C-terminal segment of Pam18 was found cross-linked to the terminal matrix loop of Tim23 (Fig. 3b).

**Fig. 7:**
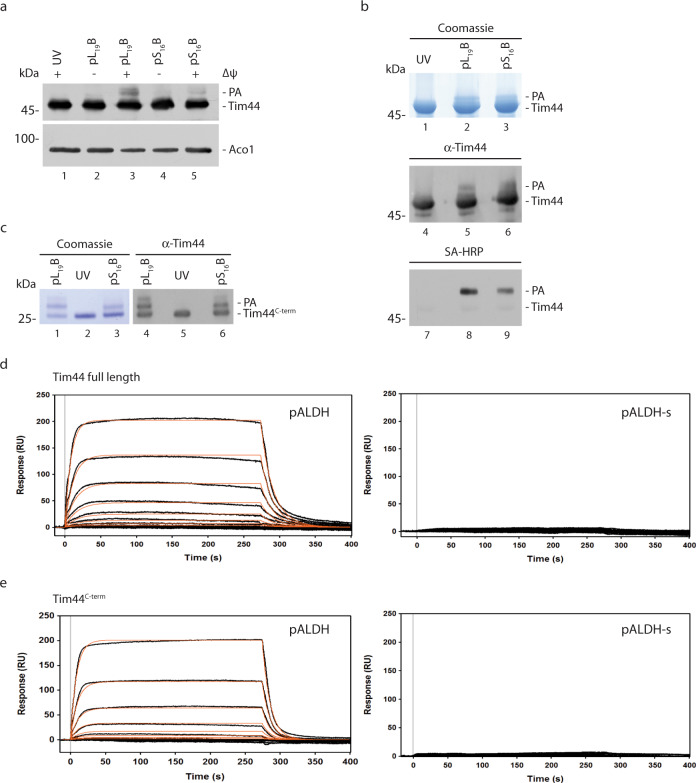
Tim44 C-terminus is involved in presequence interaction. **a** Isolated mitochondria were incubated with presequence photo-peptides pL_19_B and pL_16_B in the presence or absence of membrane potential (ΔΨ) and subjected to UV irradiation. Samples were analyzed by immunoblotting. PA: photo-adduct. Three independent replicates were carried out. Recombinantly purified **b** full length Tim44 and **c** Tim44^C-term^ were subjected to photo-peptide incubation and UV irradiation. Analysis of photo-adduct formation was carried out by SDS-PAGE and colloidal coomassie staining or immunoblotting. SA-HRP: HRP conjugated Streptavidin. Representative images from two independent experiments. Purified His_6_-tagged **d** Tim44 full length and **e** Tim44^C-term^ were analyzed by SPR (Surface Plasma Resonance) analysis at increasing analyte concentrations (0.125−16 µM). A representative sensogram is depicted. Black lines show observed binding, orange calculated *k*_*a*_/*k*_*d*_ fitting of the kinetic data. Analytes used: presequence peptide pALDH or scrambled presequence pALDH-s.

Interestingly, we also identified a cross-link between Tim44 and the matrix exposed, N-terminal portion of Jac1 downstream of the presequence (Fig. 4a and Supplementary Data 1). Already early work suggested a function of Tim44 in preproptein recognition^53–56^ and recent work confirmed an interaction between presequence peptides and Tim44^21,49^. These observations and the multiple interactions of Tim44 with motor constituents led us to assess presequence-Tim44 association further. To this end, we imported presequence peptide probes containing the photoreactive amino acid derivative para-benzoylphenylalanine (BPA) on the different faces of the helix^22^ into energized mitochondria. After UV-irradiation, we were able to detect photo-adducts of Tim44 with presequence peptides in a membrane potential-dependent manner (Fig. 7a), confirming the interaction of Tim44 with presequences in intact mitochondria during import.

To asses if the interaction between Tim44 and a presequence was direct and independent of other translocase components, we purified full-length Tim44 from *E. coli* and repeated the cross-linking approach using the photo-peptides. Coomassie-staining revealed a specific Tim44-peptide adduct that was confirmed by immunoblot using Tim44 specific antibodies and streptavidin-HRP conjugate that detects the biotinylated peptides (Fig. 7b). Tim44 contains two domains, an intrinsically disordered N-terminal domain (NTD), and a C-terminal domain (CTD) for which structural data exists^57^. To narrow down the presequence binding domain, we purified the shortened C-terminal domain (Tim44^C-term^) (aa 244-431) from *E. coli* and tested interaction with the presequence photo-peptides. Again, after UV-irradiation a specific cross-link between Tim44 CTD and presequence peptide could be visualized, indicating that the CTD binds to the presequences (Fig. 7c).

Since the cross-linking approach only allows for qualitative analysis, we used surface-plasmon resonance spectroscopy (SPR) to determine the affinity of Tim44 to presequence peptides. For this, His-tagged Tim44 was immobilized on a Ni^2+^ chelator sensor chip and presequence association was monitored over a wide range of concentrations (Fig. 7d). In line with previous results^21^, Tim44 presented a *K*_*D*_ in the lower µM range (36.25 µM) with the model peptide pALDH while there was no interaction with a scrambled peptide pALDH-s. Next, we used Tim44^C-term^ for the SPR analysis and found a slightly higher affinity to presequence peptide (15.5 µM) (Fig. 7e). Based on cross-linking analyses, previous work presented evidence that the NTD of Tim44 facilitates presequence interaction^49^. Our SPR results indicate that the CTD is able to bind presequences with similar affinity as the full-length Tim44, expanding the model of Tim44 action during precursor import. However, as the NTD of Tim44 could not be purified in a soluble form for SPR analyses, we can not exclude that both domains are capable of presequence recognition.

Over the past decades, significant advances have been made regarding the components and mechanisms of protein transport along the presequence import pathway. However, the lack of structural information on the TIM23 complex represents an obstacle for a molecular understanding of precursor transport across the intermembrane space, from the TOM to the TIM23 complex and across the inner membrane. For precursor handover from the outer to the inner membrane, a tight, presequence modulated co-operation of Tom and Tim proteins is required, yet we lack information on the topology of the constituents during translocation.

In this study, we performed biochemical and structural proteomic analyses to identify and define interaction sites in the TOM-TIM23 transition zone of the intermembrane space to understand how precursor transfer is facilitated. We utilized a strategy to specifically isolate the TOM-TIM23 supercomplex following import of an accumulated precursor, Jac1^sfGFP^. Cross-linking analyses were carried out on the isolated protein complex providing us with a large number of intermolecular cross-links (Supplementary Data 1). In addition, we utilized two complementary strategies. We performed cross-linking on intact mitochondria and purified the TOM-TIM23 supercomplex after *in organello* cross-linking. Complex purification prior to cross-linking allowed for a deeper analysis than the *in organello* approaches, probably due to the higher enrichment of Tom, Tim, and Pam proteins in the purified complex. However, the *in organello* analyses allowed us to confirm cross-links and also provided complementary sets of interactions (Supplementary Data 1 and Supplementary Table 1). Inter-protein cross-links obtained from all three approaches are summarized in Supplementary Data 2.

The cross-linking-MS analyses allowed us to position Tim23 relative to the TOM complex. These analyses revealed that the IMS domains of Tim and Tom proteins are in close proximity to support precursor handover. Different views exist in the mitochondrial import field regarding whether Tim23 spans the outer mitochondrial membrane. In the context of this long-standing controversy, our data indicate that the N-terminus of Tim23 is positioned at the intermembrane space side of the TOM complex. However, we acknowledge that these different views cannot be completely resolved based on our experimental evidence. Tim23^IMS^ also interacts with the IMS domains of Tim17, Tim50, Tim21, and Mgr2, indicating the flexibility of the Tim23 N-terminus in the intermembrane space. The topology of the TIM23 complex’ IMS domains at the Tom40 channel defines a restricted space for the incoming presequence. The proximity of receptor sites would allow for efficient precursor transfer. At the same time, considering, the relatively low affinity for presequences measured for Tim50^IMS^ and Tim23^IMS^ in solution, a high local concentration of the presequence at the Tom40 channel exit would favor receptor association. Accordingly, the TOM-TIM23 interface represents an affinity trap for presequences emerging from TOM (Fig. 6a, b). In addition to a view of interactions in the IMS, our analyses also provide insight into interactions among import motor constituents, as well as between motor constituents and the TIM23 complex. Previous work has shown that the N-terminus of Pam18 engages with the C-terminus of Tim17 in the IMS for motor recruitment^26,41^. Interestingly, we observed cross-links of Tim21^IMS^ to Tim17’s C-terminus. Accordingly, the mechanistically enigmatic switch of the translocase between TIM23^SORT^ (motor-free, TIM21 associated) and TIM23^MOTOR^^26,58^ could be linked to mutually exclusive interactions of Pam18 or Tim21 to the Tim17 C-terminus. Furthermore, an interaction of Tim50 with the scaffold protein Tim44 could explain the observed effect of Tim50 on motor-driven matrix protein import. Presequence binding of Tim44 CTD points towards a concerted mechanism of action with the NTD for protein import. To this end, our data provide an interaction map of the constituents of the active translocase that enables concerted precursor transport across two biological membranes (Supplementary Movie 1). Moreover, the atomic modeling of the TOM-TIM23 interface provided here allows us to grasp the molecular crowding of Tom and Tim proteins in the intermembrane space of the two translocase and to integrate biochemical data on the interplay between individual TOM and TIM23 components into a spatial context.

## Methods

### Yeast growth and handling

Yeast strains were grown in YP media (1% yeast extract, 2% peptone) containing 2% glucose (YPD) or 3% glycerol (YPG) as a carbon source. YPH499 (MATa *ade2-101, his3-*∆*200, leu2-∆1, ura3-52, trp1-∆63, lys2-*801, ATCC^®^ 204679™), BY4741 (MATa *his3∆1, leu2∆0, met15∆0, ura3∆0*, Euroscarf), and ^HisS*^Tim23^41^ strains were grown at 30 °C with shaking. A complete list of yeast strains generated in this study is provided in Supplementary Table 2. Jac1^sfGFP^ was expressed in BY4741 cells. For this Jac1^sfGFP^ was cloned into p425Gal1 under an inducible Galactose promoter. The resulting plasmid (pRG13) was transformed into BY4741 and transformants were selected on SSuc-Leu (selective sucrose media lacking leucine). For expression of ALFA-tagged translocase subunits, an empty plasmid with ALFA tag and ALDH terminator in pRS414 was constructed (pRG26). Subsequently, various translocase subunits with approximately 500 bp upstream promoter region were cloned 5′ of the ALFA tag, leading to the generation of plasmids as mentioned in Supplementary Table 3. Following this, the plasmids were transformed into YPH499, and transformants were selected on SD-Trp (selective glucose media lacking tryptophan). Additionally, WT Tim21 in pFL39 plasmid was transformed into Tim21^FLAG^ strain and selected on SD-Trp. For Tim23 Cysteine mutants, a Tim23 shuffling strain MB29 (*MAT*a *ade2 his3 leu2 ura3 trp1 lys2 tim23*::*LYS2* [*YCplac33*-*TIM23*]) was generated^45^, in which a *LYS2* cassette replaced the endogenous *TIM23*. This was carried out in a strain containing a plasmid expressing TIM23 with URA3 as the marker. *TIM23* gene and 1 kb upstream and downstream of the gene were cloned into pRS413 (HIS3 marker), and point mutations were introduced using site-directed mutagenesis (Supplementary Table 3). These plasmids were transformed into the shuffling strain, following which selection against Ura3 containing wild-type *TIM23* harboring plasmids was carried out on 5-Fluoroorotic acid (5-FOA). These cells were grown on YPD and YPG at 30 °C for mitochondrial preparation.

### Isolation of mitochondria

Differential centrifugation of yeast extracts was carried out to isolate mitochondria^59^. Briefly, yeast strains were cultured in respective media at 30 °C to OD_600_ of 1.5−2.5 and harvested. The pellet was washed with water, following which it was treated in DTT buffer (10 mM DTT, 100 mM Tris/HCl, pH 9.4) for 30 min at 30 °C with shaking. Subsequently, cells were washed and treated with Zymolyase buffer (20 mM KPO_4_, pH 7.4, 1.2 M sorbitol, and 0.57 mg/L zymolyase) for 1 h at 30 °C with shaking. Cells were harvested. Pellet was resuspended in cold homogenization buffer (600 mM sorbitol, 10 mM Tris/HCl, pH 7.4, 1 g/L BSA, 1 mM PMSF, and 1 mM EDTA) and cells were homogenized using a homogenizer. Differential centrifugation was carried out to obtain mitochondria. They were subsequently resuspended in SEM buffer (250 mM sucrose, 20 mM MOPS/KOH pH 7.2, 1 mM EDTA). Following protein concentration estimation by Bradford analysis, they were aliquoted in appropriate volume, flash-frozen in liquid nitrogen, and stored at −80 °C.

### Superfolder-GFP expression, Immunofluorescence staining, and STED nanoscopy of yeast cells

For fluorescence microscopy, wild-type BY4741 cells transformed with the Jac1^sfGFP^ plasmid (pRG13) were grown in SSuc-Leu media at 30 °C overnight. The next day, cells were induced with 2% galactose for 1 h. Subsequently, 0.5 µM MitoTracker^TM^ Orange CMTMRos (Thermo Fisher Scientific, MA, USA) was added to the culture. Cells were kept shaking for 20 min. Afterwards, cells were harvested, washed once with media, and analyzed using a DeltaVision fluorescence microscope (GE Healthcare, IL, USA).

For confocal and STED microscopy, yeast cells containing the pRG13 plasmid were grown in a medium containing 2% glucose as the sole carbon source. sfGFP expression was induced via the addition of 2% galactose to the medium. One hour after induction, the cells, grown to the early exponential growth phase (OD_600_ = 0.4–0.7), were fixed with formaldehyde and further treated as described previously^60^. Detection of specific epitopes was realized by incubation of the cells with antisera specific to GFP (Anti-GFP [3E6], Mouse, Thermo Fisher Scientific, Cat.no. A11120, Lot number 1859591), Tom40 and Tim23 respectively (4 °C, 16 h). Primary antibodies were detected using secondary antibodies custom labeled with Abberior STAR RED (Abberior, Göttingen, Germany) or Alexa Fluor 594 (Thermo Fisher Scientific, Waltham, MA, USA) (RT, 90 min).

STED nanoscopy was achieved using a 775 nm quad scanning STED microscope (Abberior Instruments, Göttingen, Germany). The microscope was equipped with a UPlanSApo 100×/1,40 Oil objective (Olympus, Tokyo, Japan) and excitation lasers with wave lengths of 561 nm (excitation of Alexa Fluor 594) and 640 nm (excitation of STAR RED) respectively. A pixel size of 15 nm was applied and besides contrast stretching no further image processing was applied.

### Protein expression and purification

For the expression of recombinant proteins in *E. coli*, plasmids were transformed into competent BL21 cells. For Jac1^sfGFP^ and GFP nanobody expression, cells were precultured in LB-antibiotic media overnight at 37 °C. Following this, cells were diluted to an OD_600_ of 0.1. Upon reaching OD_600_ of 0.6, protein expression was induced by treatment with 1 mM IPTG for 4 h with shaking at 37 °C for Jac1^sfGFP^ and at 30 °C for GFP nanobody. Cells were harvested. The bacterial cell pellet was lysed, following which the supernatant was applied to their respective affinity columns. For Jac1^sfGFP^, which has a 6X His tag followed by Sumo protease cleavage site at the N-terminus, HisTrap columns (GE Healthcare) were used. Purification was carried out using ÄKTA Purifier 10 (GE Healthcare). Briefly, the filtered supernatant obtained after lysis was applied to a HisTrap column pre-equilibrated with HisTrap buffer A (40 mM Tris/HCl pH 7.4, 500 mM NaCl, 10 mM Imidazole). After loading, the column was washed with HisTrap buffer A and eluted with a linear gradient of HisTrap buffer B (40 mM Tris/HCl pH 7.4, 500 mM NaCl, 500 mM Imidazole). Fractions were collected and analyzed using SDS-PAGE followed by Coomassie Brilliant Blue staining. Fractions containing protein of interest were pooled. Dialysis was carried out overnight in dialysis buffer (20 mM Tris/HCl pH 7.4, 100 mM NaCl). After dialysis, the protein solution was concentrated with Amicon centrifugal filters (Merck) with a 10 kDa cut-off column. SUMO protease treatment (1 mg protease/200 mg protein along with 1 mM DTT) was carried out overnight at 4 °C on a shaker. This was followed by size exclusion chromatography using HiLoad 16/60 Superdex 200 columns (GE Healthcare). Protein was applied to pre-equilibrated column with buffer (20 mM Tris/HCl pH 7.4, 100 mM NaCl). Fractions were collected and analyzed using SDS-PAGE and Coomassie Brilliant Blue staining. Desired fractions were pooled and following concentration estimation, protein was aliquoted, snap-frozen, and stored at −20 °C.

For GFP nanobody, GSTrap column (GE Healthcare) was used. The affinity purification procedure was the same as above, except for the buffers used (Buffer A: 140 mM NaCl, 2.7 mM KCl, 10 mM Na_2_HPO_4_, 1.8 mM KH_2_PO_4_, pH 7.3; buffer B: 50 mM Tris/HCl, 10 mM reduced glutathione, pH 8.0). After affinity purification, the protein was aliquoted and stored at −20 °C.

Purification of full length Tim44 for SPR and in vitro photo cross-linking was done as follows: *E.coli* BL21 cells expressing His_6_-tagged full length Tim44 without presequence were resuspended in cracking buffer (20 mM Tris pH 8.0, 500 mM NaCl, 15 mM Imidazole, one complete EDTA-free protease inhibitor pill, 2 mM PMSF) and lysed using Emulsiflex. The lysate was centrifuged and filtered supernatant was applied on a 1 ml HisTrap FF column. After washing with 10% buffer A, elution was carried out with a stepwise increasing concentration of buffer B (20 mM Tris pH 8.0, 500 mM NaCl, 500 mM Imidazole). Fractions containing protein were pooled and dialysis was carried out overnight at 4 °C in dialysis buffer (10 mM MOPS, 5 mM MgCl_2_, 2 mM KH_2_PO_4_, 20 mM KCl, 10 mM Imidazole, pH 7.5). For SPR analysis, protein was further subjected to size exclusion chromatography using Superdex 75 HiLoad 16/60 (Buffer: 50 mM HEPES pH 7.4, 150 mM NaCl, 50 μM EDTA).

His tagged Tim44^C-term^ for SPR: The C-terminal domain of Tim44 (residues 244−431) was cloned into a pPROEX HTc plasmid, resulting in a construct with an N-terminal His_6_ tag followed by a TEV cleavage site. Following transformation into BL21 *E. coli*, expression was obtained by inducing the cells with 1 mM IPTG for 4 h at 37 °C. Purification was carried out similar as for Tim44 full length, except the Tris buffer used was at pH 7.4. Purified protein was subjected to size exclusion chromatography using Superdex 75 HiLoad 16/60 with buffer containing 50 μM EDTA, 50 mM HEPES pH 7.4, 150 mM NaCl.

His tagged Tim44^C-term^ for in vitro photo cross-linking: The same Tim44^C-term^ construct as above was expressed in BL21 Tuner cells by inducing with 0.2 mM IPTG for 5 h at 30 °C. Following lysis in lysis buffer (50 mM HEPES pH 7.5, 300 mM KCl, 10 mM Imidazole, 5% (v/v) Glycerol, 1 mM PMSF, one complete EDTA-free protease inhibitor pill and DNaseI) using Emulsiflex, cleared lysate was applied to a HisTrap column. Protein was eluted with a linear gradient of elution buffer (50 mM HEPES pH 7.5, 300 mM KCl, 300 mM Imidazole, 5% (v/v) Glycerol). Pooled fractions containing protein were concentrated and subjected to size exclusion chromatography (Buffer: 50 mM HEPES pH 7.5, 200 mM KCl, 5% (v/v) Glycerol) using Superdex 75 HiLoad 16/60. Fractions containing protein were pooled and protein concentration was determined using Bradford assay.

### Import of precursor proteins and generation of the TOM-TIM23 supercomplex

For the synthesis of [^35^S] methionine-labeled F1β protein, mMessagemMachine SP6 transcription kit (Invitrogen, CA, USA) was used first to generate mRNA in vitro based on the manufacturer’s instructions. Subsequently, in vitro translation was carried out using the Flexi Rabbit Reticulocyte Lysate System (Promega, WI, USA). Prepared lysates were used directly for import reactions.

Import of radiolabelled precursor or recombinant protein was performed as previously described^61^. Briefly, mitochondria were suspended in import buffer (250 mM sucrose, 10 mM MOPS/KOH pH 7.2, 80 mM KCl, 2 mM KH_2_PO_4_, 5 mM MgCl_2_, 5 mM methionine, and 3% fatty acid-free BSA) supplemented with 2 mM ATP, 2 mM NADH, 5 mM creatine phosphate and 0.1 mg/ml creatine kinase, to have a final concentration of 1 µg/µl. Import was performed for desired time points (30 min for Jac1^sfGFP^, 15 min for [^35^S] F1β) at 25 °C under mild shaking and was terminated by addition of 1% AVO (final concentration 1 µM valinomyin, 8 µM antimycin A and 20 µM oligomycin). Non-imported precursors were digested by 20 µg/ml proteinase K (PK) treatment for 10 min, whenever required. PK reaction was inactivated by 2 mM PMSF. Mitochondria were subsequently sedimented and washed with SEM buffer. Samples were analyzed by SDS-PAGE or BN-PAGE in combination with western blotting or autoradiography. After digital autoradiography, quantifications were performed using ImageQuant TL (GE Healthcare, NJ, USA) using a rolling ball background subtraction. Results were calculated as mean ± SEM, *n* = 4 and graphically represented using GraphPad Prism 8.

For import arrest assay, indicated amounts of recombinantly purified Jac1^sfGFP^ were imported into mitochondria for 30 min. Mitochondria were then sedimented and resuspended in fresh import buffer. This was followed by the import of 6% ^35^S-labeled F1β for 15 min. After this, mitochondria were treated as described above, and analyzed by SDS-PAGE and autoradiography.

### Cysteine modification assay

Mitochondria and mitoplasts at a concentration of 1 mg/ml were generated by incubating isolated mitochondria from Tim23 WT and Cysteine mutants in SEM (250 mM sucrose, 20 mM MOPS/KOH pH 7.2, 1 mM EDTA) and EM (20 mM MOPS/KOH pH 7.2, 1 mM EDTA) buffer respectively for 15 min on ice. Subsequently, they were treated with Maleimide activated Streptavidin (Thermo Fisher Scientific) at a final concentration of 1 mg/ml and incubated for 30 min on ice followed by 60 min at 25 °C. Samples were harvested by centrifugation and resuspended in protein loading dye containing beta-mercaptoethanol. Following this, they were boiled at 95 °C for 5 min and analyzed by NuPAGE^TM^ 4−12% Bis-Tris gels and immunoblotting using Tim23^IMS^ antibody. The images for Tim23^T9C^ were captured digitally (Amersham™ ImageQuant™ 800) due to the weak detection of Tim23^T9C^ by this antibody with X-ray films.

### Photo cross-linking assay

*In organello* photo cross-linking was performed as previously described^22^. Briefly, mitochondria were resuspended to a concentration of 1 mg/ml in import buffer without BSA, supplemented with 1 mM ATP and 1 mM NADH. AVO was added for membrane potential controls. After incubation for 2 min at 25 °C, photo-peptides pL_19_B and pS_16_B were added to a final concentration of 2 μM and incubated for 10 min on ice. Subsequently, photo-peptides were cross-linked for 30 min on ice using a halogen metal vapor lamp and a glass screen to protect the proteins from high-energy radiation. Following centrifugation and washing with SEM buffer, mitochondria were resuspended in protein loading dye and analyzed by SDS PAGE and western blotting.

For in vitro photo cross-linking, purified full length Tim44 was mixed in equimolar ratio with presequence peptides pL_19_B and pS_16_B or 10 mM acetic acid, incubated for 10 min on ice, and cross-linked for 30 min on ice. Photo-adducts were analyzed by SDS-PAGE, colloidal coomassie staining, and immunoblotting (Peroxidase Streptavidin, Jackson ImmunoResearch Lab, Cat. No. 016-030-084). For Tim44^C-term^, the purified protein in 25 mM HEPES pH 7.5, 100 mM KCl, 5 mM MgCl_2_ buffer was incubated with equimolar ratio presequence peptides. Cross-linking and analysis were carried out the same as above.

### Glycerol density gradients

10−30% glycerol gradients were prepared by mixing 10% glycerol buffer (10% glycerol, 20 mM Tris/HCl, pH 7.4, 100 mM NaCl, 5 mM EDTA, 0.3% digitonin) and 30% glycerol buffer (30% glycerol, 20 mM Tris/HCl, pH 7.4, 100 mM NaCl, 5 mM EDTA, 0.3% digitonin) using pre-programmed conditions for 10−30% glycerol gradients on Gradient Master (BioComp Instruments). They were subsequently cooled at 4 °C for 2−3 h. Solubilized mitochondria, after being incubated with buffer or Jac1^sfGFP^ for 30 min, were overlayed on the gradients, after which ultracentrifugation was carried out in Sw60Ti rotors (Beckmann Coulter) for 18 h at 121,262 × *g* at 4 °C. Fractions were collected from the top and were analyzed by SDS-PAGE followed by immunodecoration. Signals were quantified using ImageQuant TL.

### Isolation of complexes

Two different strategies were utilized for the isolation of protein complexes: 1) Using GFP nanobody to isolate the TOM-TIM23 supercomplex generated by the import of Jac1^sfGFP^, or 2) via TIM23 complex isolation using His-SUMOstar tag on Tim23.

In the first approach, following the generation of the TOM-TIM23 supercomplex, mitochondria were sedimented, washed with SEM, and solubilized in solubilization buffer (20 mM HEPES/KOH pH 7.4, 150 mM NaCl, 10% glycerol, 0.1 mM EDTA, 1% digitonin, 1 mM PMSF). Strep-tactin sepharose beads (IBA) were equilibrated with water and PBS. The beads were incubated with purified GFP nanobody for 1 h at room temperature, on shaking. Subsequently, the beads were washed with 2× buffer (40 mM HEPES/KOH pH 7.4, 300 mM NaCl, 40% glycerol, and 0.2 mM EDTA) and solubilization buffer. The clarified supernatant of solubilized mitochondria was applied to the beads and binding was carried out for 1 h at 4 °C. Following this, the beads were washed with a buffer containing 0.3% digitonin. Elution was carried out with 7.5 mM desthiobiotin. Samples were analyzed by SDS-PAGE and western blotting (anti-GFP, Roche, Cat. No. 11814460001). The total was always loaded at 5% of elution unless otherwise indicated.

For the TIM23 complex isolation, ^HisS*^Tim23 yeast strains were utilized. In these strains, the endogenous Tim23 has been replaced by ^HisS*^Tim23. Mitochondria from this strain were incubated with buffer or Jac1^sfGFP^. Ni^2+^-NTA agarose beads (Macherey Nagel) were washed with 2× buffer and solubilization buffer. Solubilized mitochondria were incubated with equilibrated beads for 2−3 h at 4 °C under mild shaking. Beads were washed with buffer without PMSF, and elution was carried out using 1 µM SUMOstar protease (kindly provided by Dr. Alexander Stein) in wash buffer without PMSF for 1 h at 4 °C. For mitochondria that were subjected to Jac1^sfGFP^ import, a second GFP nanobody isolation step can be added after isolation of the TIM23 complex. This two-step isolation strategy leads to a specific isolation of the TOM-TIM23 supercomplex. Samples obtained after isolation were assessed by SDS-PAGE and western blotting or directly subjected to cross-linking-MS analysis.

For analysis of copy number of mitochondrial translocase subunits, ALFA IP and FLAG IP was carried out. Briefly, mitochondria from yeast cells expressing endogenous and tagged proteins for Tim23, Tim50, Tim17, Tim44, Pam16, Pam18, and Tim21 were solubilized in 1% digitonin solubilization buffer as described previously. Clarified extracts were incubated with equilibrated ALFA Selector^ST^ (NanoTag Biotechnologies) or FLAG beads for 1 h at 4 °C. After few rounds of washing, elution was carried out using SDS Protein loading dye. Samples were analyzed by SDS-PAGE and western blotting (anti-ALFA, NanoTag Biotechnologies, Cat. No. N1502-HRP; anti-FLAG, Sigma, Cat. No. F3165). Images were processed using ImageJ v1.47. A complete list of antibodies used in this study along with their dilution is provided in Supplementary Table 4.

### Surface plasmon resonance—SPR

The binding of pALDH and pALDH-s to various 6xHis-tagged Tim44 ligands was analyzed using surface plasmon resonance. SPR experiments were performed on a Reichert SPR Biosensor (SR700DC, Xantec Bioanalytics, Düsseldorf, Germany) equipped with a Ni2+-chelator sensorchip (NiHC500m, Xantec Bioanalytics, Düsseldorf, Germany) in running buffer (50 mM HEPES, pH 7.4, 150 mM NaCl, 50 µM EDTA) at 20 °C. The ligands were diluted in running buffer to final concentrations of 200 nM and injected over the Ni2+-activated left side of a SPR sensor chip surface at a flow rate of 30 µL/min to final responses of ~2500 µRIU (micro refractive index units). The right channel was used as a reference channel and left unmodified. The analytes were serially diluted in running buffer (16, 8, 4, 2, 1, 0.5, 0.25, and 0.125 µM) and injected over both channels at a flow rate of 40 µL/min. The association and dissociation of each analyte were followed for 4.5 and 7 min, respectively. Two buffer injections were performed per analyte and used as buffer reference. The obtained response data were analyzed with and kinetic and equilibrium binding parameters were determined using Scrubber 2.0 (BioLogic Software). Each data set was double referenced (reference channel, buffer injections/buffer reference). Experiments were essentially conducted as described before^25^.

### Cross-linking analysis

For SDS-PAGE analysis of cross-linking, a two-step supercomplex isolation was carried out from ^HisS*^Tim23 mitochondria following Jac1^sfGFP^ import. The isolated complex was treated with DMSO, 2 mM DSS, 2 mM DSSO, and 10 mM EDC for 2 h on ice. Cross-linking reaction was quenched with 250 µM Glycine pH 8.0 for 30 min on ice. Samples were analyzed using western blotting. Additionally, to analyze cross-links between Tim21 and Tom22, ^HisS*^Tim23 and ^HisS*^Tim23 *tim21∆* mitochondria were subjected to Jac1^sfGFP^ import followed by TIM23 complex isolation. Elution fractions were treated with DMSO or 2 mM DSS, quenched with glycine, and analyzed by SDS-PAGE and immunoblotting.

For TIM23 complex isolation followed by cross-linking and mass spectrometry (Approach 1), the TIM23 complex was isolated after the import of buffer or Jac1^sfGFP^. The isolated complex was treated with 2 mM DSS or 10 mM EDC for 3 h on ice, followed by quenching of the reaction with 250 µM glycine pH 8.0 for 30 min. Proteins were precipitated overnight at −20 °C using 80% acetone. The following day, samples were centrifuged. For SDA cross-linking, 2 mM SDA (Thermo Fisher Scientific) was added to the isolated complex for 2 h at 4 °C. The cross-linking reaction was quenched with 50 mM Tris-HCl and the proteins were dialyzed against reconstitution buffer via a membrane filter (MF Membrane Filters, 0.025 µm VSWP, Merck). Afterwards, the samples were irradiated with UV light (365 nm) for 5 min at 4 °C.

For *in organello* cross-linking (Approach 2), ^HisS*^Tim23 mitochondria were subjected to incubation with buffer or Jac1^sfGFP^. Following centrifugation and washing with HN buffer (20 mM HEPES pH 7.4, 100 mM NaCl), mitochondria were resuspended in HN buffer and subjected to cross-linking with 1 mM DSS or 5 mM EDC/10 mM Sulfo NHS for 3 h on ice. The reaction was quenched with 50 mM Tris pH 8.0 or 50 mM Tris pH 8.0/20 mM DTT for DSS and EDC samples respectively. Samples were centrifuged and analyzed by mass spectrometry.

For import of Jac1^sfGFP^ followed DSS cross-linking and TIM23 complex isolation (Approach 3), ^HisS*^Tim23 mitochondria were incubated with buffer or Jac1^sfGFP^. Harvested mitochondria were resuspended in HN buffer and cross-linked with 1 mM DSS for 3 h on ice. The reaction was quenched using 50 mM Tris pH 8.0, and TIM23 complex isolation was carried out as described previously. The isolated complex was precipitated using acetone and subjected to mass spectrometry analysis.

### Protein digestion and enrichment of cross-linked peptides

Cross-linked proteins were resuspended in 4 M urea/50 mM ammonium bicarbonate, reduced with 10 mM dithiothreitol, and subsequently alkylated with 40 mM iodoacetamide. Proteins were digested with the endoproteinase trypsin in an enzyme-to-protein ratio of 1:50 in the presence of 1 M urea at 37 °C overnight. The reaction was terminated with 0.5% trifluoroacetic acid (TFA) (v/v) and peptides were desalted on MicroSpin Columns (Harvard Apparatus) following the manufacturer’s instructions. Vacuum-dried peptides were resuspended in 50 µL 30% acetonitrile/0.1% TFA. Cross-linked peptides were enriched by peptide size exclusion chromatography (SuperdexPeptide 3.2/300 column, GE Healthcare)^62^ or basic pH reversed-phase chromatography (for SDA samples). Fractions of 50 µL were collected. Early eluting fractions that contain cross-linked peptides were subjected to LC-MS/MS analysis.

For *in organello* cross-linking (Approach 2): DSS or EDC cross-linked mitochondria were lysed with 2% SDS, precipitated at −80 °C for 2 h, digested with trypsin overnight in the presence of 1 M Urea and enriched for cross-linked peptides by peptide SEC. The first seven fractions were then measured with a 3 h method, and, additionally, these fractions were pooled and separated via basic RP. From that, 24 fractions were collected, and also measured.

For Approach 3, cross-linked complexes were precipitated with acetone, pelleted by centrifugation, dissolved in 50 mM ammonium bicarbonate (pH 8.0) supplemented with 6 M Urea, reduced with tris(2-carboxyethyl)phosphine (TCEP), and alkylated with chloroacetamide. After dilution to 1 M urea with 50 mM ammonium bicarbonate, cross-linked complexes were digested with trypsin (Promega) in a 1:20 enzyme-to-protein ratio (w/w) at 37 °C overnight. Peptides were reverse-phase extracted using SepPak Vac tC18 1cc/50 mg (Waters) and eluted with 50% acetonitrile (ACN) / 0.1 % trifluoroacetic acid (TFA). The eluates were lyophilized, dissolved in 40 µl 30% ACN / 0.1% TFA and subjected to peptide size exclusion chromatography (pSEC, using a Superdex Peptide PC3.2/300 column, GE Healthcare) at a flow rate of 50 µl/min using Agilent 1200 Series HPLC system. Fractions of 50 µl were collected.

### LC-MS/MS analysis

LC-MS/MS analyses were performed as described elsewhere^63^. Briefly, peptides cross-linked by DSS were measured in technical duplicates on an Orbitrap Fusion or Fusion Lumos Tribrid Mass Spectrometer coupled to a Dionex UltiMate 3000 UHPLC system (both Thermo Fisher Scientific) equipped with an in house-packed C18 column (ReproSil-Pur 120 C18-AQ, 1.9 µm pore size, 75 µm inner diameter, 30 cm length, Dr. Maisch GmbH). MS1 full scans were acquired in the orbitrap (OT) with a resolution of 120,000, an injection time (IT) of 60 ms, and an automatic gain control (AGC) target of 5 × 10^5^. Dynamic exclusion (DE) was set to 10 s and only charge states between 3 and 8 were considered for fragmentation. MS2 spectra were acquired in the OT of the 20 most abundant precursor ions; the resolution was set to 30,000; the IT to 120 ms and the AGC target to 5 × 10^4^. Fragmentation was enforced by higher-energy collisional dissociation (HCD) at 30% NCE.

Peptides cross-linked with EDC and SDA were measured with the following changes: a Q Exactive HF-X Mass Spectrometer and Q Exactive HF Mass Spectrometer (Thermo Fisher Scientific) respectively were used; IT was set to 50 ms and AGC target to 1×10^6^ at MS1 level. At MS2 level, IT was set to 128 ms and AGC target to 1 × 10^5^. DE covered 30 ms. The 30 most abundant precursor ions were considered for fragmentation.

For analysis of cross-linked peptides from approach 3, fractions enriched in cross-linked peptides were vacuum dried, dissolved in 5% ACN / 0.1% TFA, and subsequently analyzed in duplicates in a Thermo Orbitrap Fusion Tribrid mass spectrometer coupled to a Dionex UltiMate 3000 uHPLC system (Thermo Scientific) with a custom 30 cm C18 column (75 µm inner diameter packed with ReproSil-Pur 120 C18-AQ beads, 1.9 µm pore size, Dr. Maisch GmbH). MS1 and MS2 resolution were set to 120,000 and 30,000, respectively. Only precursors with a charge state of 3−8 were triggered for MS2.

### Data analysis

Raw files that were acquired for samples cross-linked with DSS were converted to mgf file format by Proteome Discoverer (v. 1.4, Thermo Fisher Scientific). The signal-to-noise ratio was set to 1.5 and the precursor mass between 1,000 and 10,000 Da. The mgf files were analyzed by pLink 1 (v. 1.23) for the identification of cross-linked peptides^64^. Default settings were applied with carbamidomethylation of cysteine residues as fixed modification and oxidation of methionine residues as variable modification. The false discovery rate (FDR) was set to 1% at the spectrum level. A dedicated protein database including known proteins of the TOM-TIM23 complex was provided for the search of samples including the precursor. For samples without precursor, protein databases contained all identified proteins based on the identification of linear peptides by MaxQuant (v. 1.6.0.1)^65^. Cross-linked peptide spectrum matches (CSMs) were evaluated manually. Cross-linking results were visualized by xiNET^66^.

Raw files that were acquired for samples cross-linked with EDC and SDA were analyzed by pLink 2 (v. 2.3.9) for the identification of cross-linked peptides^67^. The following changes were applied compared to the described analysis by pLink 1: FDR was set below 5% on spectrum level; CSMs were not evaluated manually.

Searches for files obtained from *in organello* (Approach 2) cross-linking were performed by pLink2, against the dedicated TOMTIM database, and also against the top300 most abundant proteins that were identified in the samples (plus the dedicated TOMTIM proteins included).

Approach 3 protein-protein cross-links were identified by pLink (v. 2.3.9) search engine (pfind.ict.ac.cn/software/pLink)^64,67^ using either a complete SwissProt *S. cerevisiae* database or a custom database containing 125 proteins most abundantly present in the samples. The results are shown after filtering at FDR of 1% (more stringent) or 5%.

### Completion of the *S. cerevisiae* TOM40 complex atomic model

The *S. cerevisiae* TOM complex (PDB id: 6JNF, EMDB id: 9851) was completed by rebuilding a few missing Tom40 loops with ROSETTA tools^68^ utilizing a cryo-EM map as spatial restrains. Both the deposited as well as filtered to 4.0 Å resolution cryo-EM map (Supplementary Fig. 4a, b) were used. The missing C-terminal tail of Tom22 (residues 131−152, in total 22) was built using the ab initio folding protocol as implemented in ROSETTA^52^. For this purpose, over 350,000 decoys were obtained of the 32-residue long C-terminal tail (residues 121−152). Additional 10 residues, proceeding the missing sequence, were added to increase the chance of obtaining C-terminal conformations compatible with the structurally characterized part, which could later be used to superpose the known part of Tom22 (residues 121−130) with the newly folded fragment. The best 1000 ab initio models, according to the ROSETTA overall score, were subjected to clustering analysis using MaxCluster software (http://www.sbg.bio.ic.ac.uk/maxcluster). Clustering utilized the pairwise nearest neighbor (PNN) method employing the all-versus-all pairwise calculation of the RMSD (Root Mean Squared Deviation) and resulted in the assignment of 899 decoys into 26 clusters at a threshold of 0.55 Å. The final ab initio model was chosen from a cluster comprising the largest number of best scoring decoys (the lowest energy cluster, total size 133 decoys, spread 0.472 Å). A few lowest energy decoys (1st, 5th, 10th) constituting that cluster were manually inspected in Coot^69^ and superimposed with the structurally characterized C-terminal fragment of Tom22 (residues 120–131, PDB id: 6JNF). The decoy with the best overall fit (the 10th best according to ROSETTA score) was used as a template for restoring the missing 22 residues long C-terminus of Tom22.

### Atomic models of IMS Tim components

The atomic model of the mitochondrial J-domain-containing protein Jac1 (residues 1−47 of chain A, PDB id: 3UO3) was manually remodeled in Coot so that its N-terminal fragment adopted an extended conformation which could easily pass through the Tom40 β-barrel. No secondary structure elements were changed (only loop fragments). Structural models of Sc. Tim21 (residues 100−225, PDB id: 2CIU) and Sc. Tim50 (residues 162−361, PDB id: 3QLE) as well as the NMR structure of Sc. Tim23 (homodimer, residues 1−222 of chains A and B, PDB id: 7CLV) were downloaded from the PDB. The atomic model of Tim23 revealed a dynamic nature (a very high flexibility) of its sixty residue long N-terminal tail in solution. None of the 15 deposited conformers were satisfying observed cross-links, hence the 60 amino acid long N-terminal tail was modeled using the cross-link guided molecular modeling protocol^70^ utilizing ROSETTA ab initio folding^52^. The cross-link-derived spatial constraints were incorporated as a flat harmonic function. It guaranteed that models were penalized only if the Euclidean distance between two cross-linked atoms exceeded the specified threshold. In case of DSS cross-links (K8-K32, K32-K25) obtained for Tim23 complex without the blocking peptide, the CA-CA and CB-CB thresholds were set to 30 and 22 Å, respectively. Additional shorter EDC-derived cross-link-based spatial constraints (10 Å: M1-D12, M1-D13; 14 Å: D22-K27) were used for calculating one-third of decoys. The difference in applied length of spatial constraints derived from EDC cross-link results from different individual atoms selected for distance restraints and being at the same time present in the low-resolution ab initio step. From over 421,000 ab initio decoys, 5000 models with the lowest ROSETTA score were analyzed in order to select those which satisfy DSS and EDC cross-link derived spatial restraints. These decoys were subjected to clustering analysis using the MaxCluster program. The final model comprising 60 N-terminal amino acids of Sc. Tim23 (number 2, the second-best decoy according to ROSETTA score) was selected from the cluster comprising the highest number of lowest energy decoys (the lowest energy cluster). Prior to docking with ROSETTA^71^, two copies of the N-terminal fragment, one for each chain, were manually placed in the close vicinity of the remaining part of Tim23 (conformer 12) to satisfy cross-link-based restraints: DSS: K66-K25, K66-K32. This allowed us to skip the low-resolution global docking step and utilize a high-resolution docking ROSETTA protocol^72^. The docking was performed sequentially. Upon placement of the first N-terminal fragment, the second was docked to the model comprising the already placed one. For each docking, at least 100,000 decoys were generated using standard settings as implemented in ROSETTA high-resolution local docking protocol. No positional restraints derived from cross-link experiments were applied during docking calculations. These restraints were used only for validation purpose. It should be noted that ROSETTA docking protocol did not result in a significant repositioning of the docked molecules when compared to their initial positions. Calculated RMSDs were below 1 Å and 3 Å for the best scoring (I_sc) models of the first and the second docked N-terminal fragment, respectively. The best 100 decoys, as ranked based on I_sc, were inspected in Pymol and their differences in position were assessed by calculating the RMSD against the decoy with the lowest I_sc score (target molecule). No superposition was applied. In the case of the first docked N-terminal fragment, 90 decoys revealed the RMSD difference lower than 0.25 Å, while in the case of the second N-terminal fragment, only six decoys formed a cluster with the maximal RMSD of 0.45 Å. These two docked N-terminal fragments, with the lowest interface scores (I_sc), were used to replace coordinates of the respective fragments of the 12th conformer of Sc. Tim23 structure (chains A and B, residues 1−60). In order to provide a more complete assembly of IMS Tim components, the missing atomic model of the C-terminal domain of Sc. Tim50 (residues 348−476) was modeled using the aforementioned cross-link guided molecular modeling protocol^70^ utilizing ROSETTA ab initio folding^52^. Three sets of at least 150,000 decoys were generated employing different amounts of cross-link derived spatial restraints. Set 1 was generated using pure ab initio folding protocol (without any spatial restraints). Sets number 2 and 3 comprised decoys calculated using eight and ten (maximum amount) cross-link derived restraints, respectively. For each set, 5000 best decoys were selected based on the ROSETTA overall score and subjected to an analysis yielding those which satisfy cross-link derived restraints (aforementioned distance thresholds were used for DSS cross-links). Out of these decoys, three representative sets of 26 models, selected based on the lowest ROSETTA overall score, were subjected to clustering analysis using MaxCluster. Clustering employing the nearest neighbor clustering method assigned all 78 decoys into 1 cluster at a threshold of 0.8 Å (cluster spread 0.69 Å). The final ab initio model of the C-terminal domain of Sc. Tim50 was chosen based on the ROSETTA score and belonged to set 1 (pure ab initio modeling). This decoy was superposed with the structurally characterized domain of Sc. Tim50 based on an α-helical fragment present in both the crystal structure (PDB id: 3QLE) as well as the ab initio model (residues 339−358). Superposition of these two domains satisfied experimentally obtained DSS cross-link-based spatial restraints between two Tim50 domains (K281-K348 and K288-K349). The relative position of the manually assembled domains of Sc. Tim50 was optimized using the high-resolution docking protocol as implemented in ROSETTA^72^ without using any cross-link-based distance restraints. Out of 10,000 decoys, 100 models, selected based on the I_sc score, were inspected in Pymol, and differences in their relative positions were analyzed using the aforementioned approach (calculation of the RMSD against the decoy with the lowest I_sc score). This analysis revealed that calculated RMSD between Cα atoms comprising the docked C-terminal Sc. Tim 50 domain was lower than 2.0 Å for 80 decoys while 11 of these were positioned within the distance of 1.0 Å when compared to the target model. The completed Sc. Tim50 model comprising the crystal structure (PDB id: 3QLE) and the docked C-terminal ab initio folded domain was used for building an assembly of TOM-TIM23 components.

### Modeling of the TOM-TIM23 assembly

Prepared atomic models of Tim50 components were manually assembled in Pymol relative to the completed TOM complex in order to satisfy the highest number of cross-link derived inter-molecular spatial restraints and to maximize the compactness of the whole assembly. Subsequently, the position of individual components (Tim21, Tim50) was sequentially optimized using the aforementioned ROSETTA docking protocol. The position of Sc. Tim23 relative to the Sc. TOM complex was not optimized by ROSETTA docking approach since the observed cross-links were formed between flexible fragments of two compartments anchored to membranes separated by the IMS and forming most likely only a transient interaction. Docking calculations were performed without any positional restraints. 100 decoys selected based on their I_sc score were inspected in Pymol and clustered based on their RMSD calculated without superposition against the best-scored model (target). Initially, the Tim21 structure was docked to Tom22 (19,000 decoys). RMSD based analysis revealed that seven decoys were positioned almost identically (RMSD calculated relative to the target molecule was lower than 0.5 Å), while the remaining 91 decoys revealed RMSD lower than 6 Å. Finally, the Sc. Tim50 was docked to the TOM-Tim21-Tim23 assembly (119,000 decoys). When compared to the target decoy, 5 decoys revealed RMSD difference below 0.5 Å, while in total 96 exhibited RMSD difference below 1.9 Å. The optimized assembly of Tim50-TOM IMS components was verified using cross-link-based restraints. It should be noted that the modeled assembly is only a snapshot of a highly dynamic system, and could be potentially incomplete in terms of participating components. The main aim of the undertaken modeling approach was to visualize the crowdedness of the IMS space using available atomic models, which reflect both the size and dimensions of the used compartments. Necessary manual model adjustments were performed in Coot^69^. Figures were made with Pymol (www.pymol.org).

### Reporting summary

Further information on research design is available in the Nature Research Reporting Summary linked to this article.

## Supplementary information

Supplementary Information

Dataset 1

Dataset 2

Supplementary Movie 1

Description of additional supplementary files

Reporting Summary

## Data Availability

The following structures available on the Protein Data Bank (PDB) were utilized for modeling: the TOM complex, PDB id: 6F; Jac1, PDB id: 33; Tim21, PDB id: 2U; Tim50, PDB id: 3E and Tim23, PDB id: 7V. Data and mass spectrometry datasets supporting the findings of this manuscript are available within the paper, supplementary data, and the source data files. The cross-linking mass spectrometry data generated in this study have been deposited to the ProteomeXchange Consortium (www.proteomexchange.org) via the PRIDE partner repository under accession code PXD028002. Additional information is available from the corresponding author upon request. Source data are provided with this paper.

## Source data

Source Data
