# Supplementary material for: Mapping protein interactions in the active TOM-TIM23 supercomplex
Source: Nat Commun. 2021 Sep 29;12:5715. doi: 10.1038/s41467-021-26016-1 (PMC8481542; doi:10.1038/s41467-021-26016-1)
Supplement: Supplementary file 1 — Supplementary Information [file 41467_2021_26016_MOESM1_ESM.pdf]

SUPPLEMENTARY INFORMATION

Mapping protein interactions in the active TOM-TIM23 supercomplex

Ridhima Gomkale[1], Andreas Linden[2,3], Piotr Neumann[4], Alexander Benjamin Schendzielorz[1], Stefan Stoldt[5,6], Olexandr Dybkov[7], Markus Kilisch[8], Christian Schulz[1], Luis Daniel Cruz-Zaragoza[1], Blanche Schwappach[8], Ralf Ficner[4,9], Stefan Jakobs[5,6,9], Henning Urlaub[2,3], Peter Rehling[1,9,10*]

1 Department of Cellular Biochemistry, University Medical Center Göttingen, 37073 Göttingen, Germany
2 Department of Clinical Chemistry, University Medical Center Göttingen, 37075 Göttingen, Germany
3 Bioanalytical Mass Spectrometry Group, Max Planck Institute for Biophysical Chemistry, Am Fassberg 11, 37077 Göttingen, Germany
4 Department for Molecular Structural Biology, Georg-August-Universität Göttingen, 37077 Göttingen, Germany
5 Department of NanoBiophotonics, Max Planck Institute for Biophysical Chemistry, 37077 Göttingen, Germany
6 Clinic of Neurology, University Medical Center Göttingen, 37075 Göttingen, Germany
7 Cellular Biochemistry, Max Planck Institute for Biophysical Chemistry, Am Fassberg 11, 37077 Göttingen, Germany
8 Department of Molecular Biology, University Medical Center Göttingen, 37073 Göttingen, Germany
9 Cluster of Excellence "Multiscale Bioimaging: from Molecular Machines to Networks of Excitable Cells" (MBExC), University of Göttingen, Germany
10 Max Planck Institute for Biophysical Chemistry, 37077 Göttingen, Germany

***** Corresponding author and lead contact: peter.rehling@medizin.uni-goettingen.de

Content:

Supplementary Figures 1 to 4

Supplementary Tables 1 to 4

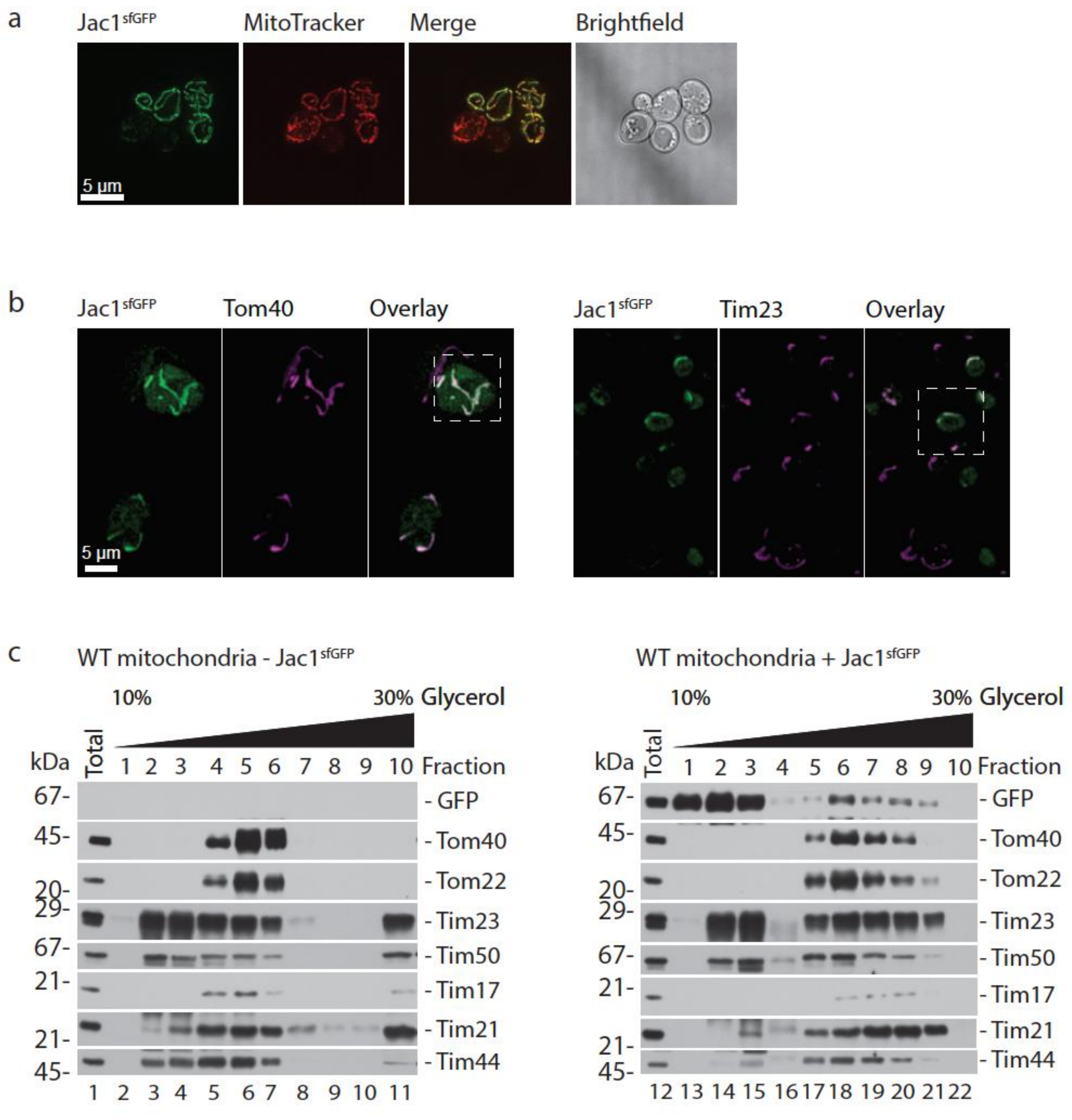

Supplementary Fig. 1. Supercomplex-forming Jac1^{sfGFP} localizes to mitochondria. a Yeast cells were transformed with Jac1^{sfGFP} (green)-encoding plasmid. After induction of expression, cells were co-stained with MitoTracker^{TM} Orange CMTMRos (red). Merged green and red fluorescence images are shown, indicating co-localization (yellow). (Scale bar = 5 µm). Representative images from three independent experiments are depicted. **b** Two-colour confocal microscopy of yeast cells expressing Jac1^{sfGFP}. Cells were labelled with antibodies against GFP (green) and Tom40 (magenta) or Tim23 (magenta). (Scale bar, 5 µm). Representative images from three biological replicates are depicted. **c** Solubilized wild type mitochondria were fractionated on 10-30% glycerol gradients following import of Jac1^{sfGFP}. Fractions were collected from top, TCA precipitated, and analyzed by SDS-PAGE followed by western blotting. Total: 2% of mitochondria loaded on the gradient. Representative experiment from a biologic triplicate is shown.

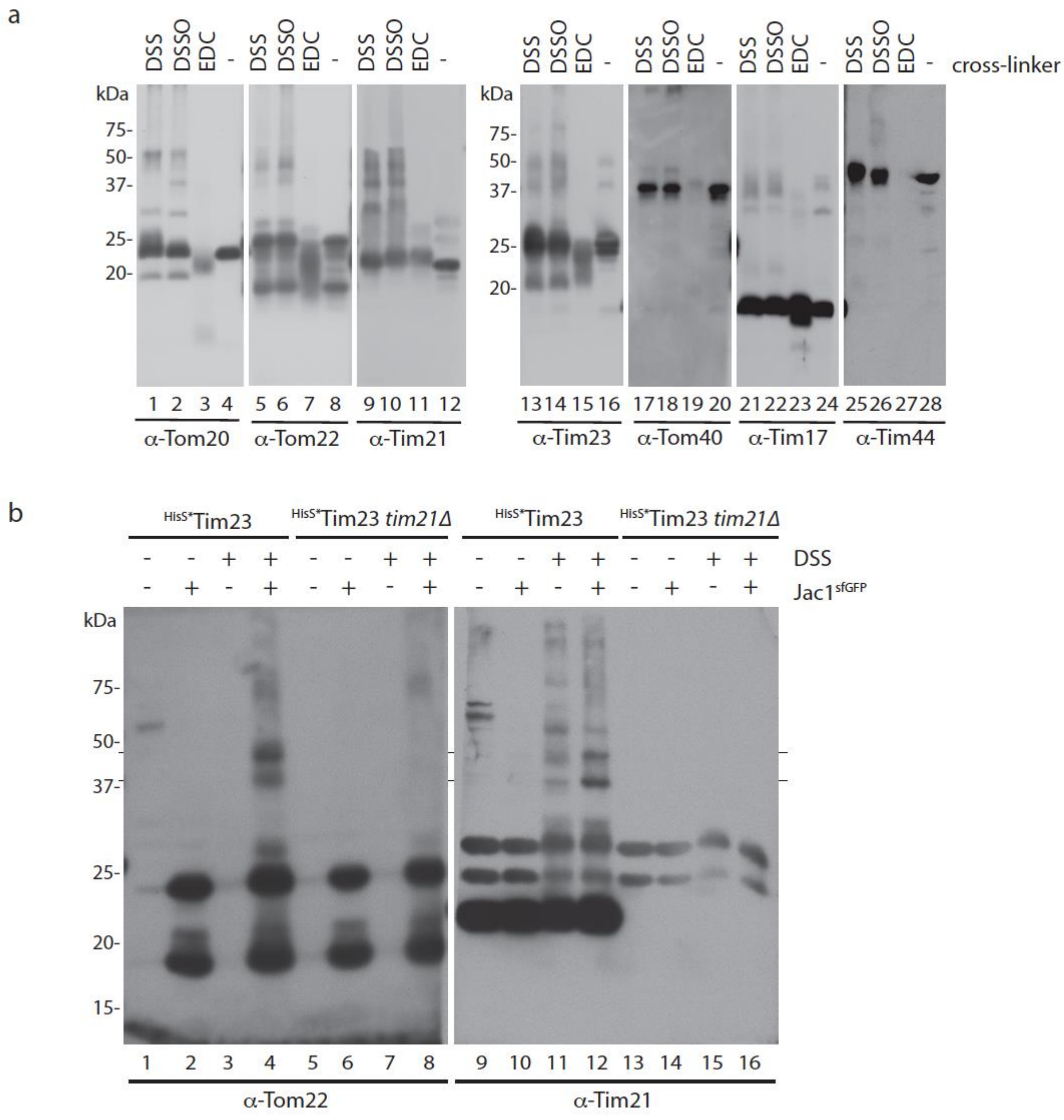

Supplementary Fig. 2. Chemical cross-linking of supercomplex subunits. a Supercomplex isolation was carried out from ^{HisS*}Tim23 mitochondria subjected to Jac1^{sfGFP} import. Isolation was performed using SUMO* protease. Isolated complex was treated with DMSO, 2 mM DSS, 2 mM DSSO and 10 mM EDC. Samples were analyzed by SDS-PAGE and western blotting. DSS: disuccinimidyl suberate, DSSO: disuccinimidyl sulfoxide, EDC: 1-Ethyl-3-(3-dimethylaminopropyl)carbodiimide. Three independent experiments were carried out. **b** TIM23 complex was isolated from ^{HisS*}Tim23 or ^{HisS*}Tim23 *tim21Δ* mitochondria in the absence or presence of Jac1^{sfGFP}. Eluted samples were treated with DMSO or 2 mM DSS. Samples were analyzed by SDS-PAGE and western blotting. Three replicates were carried out.

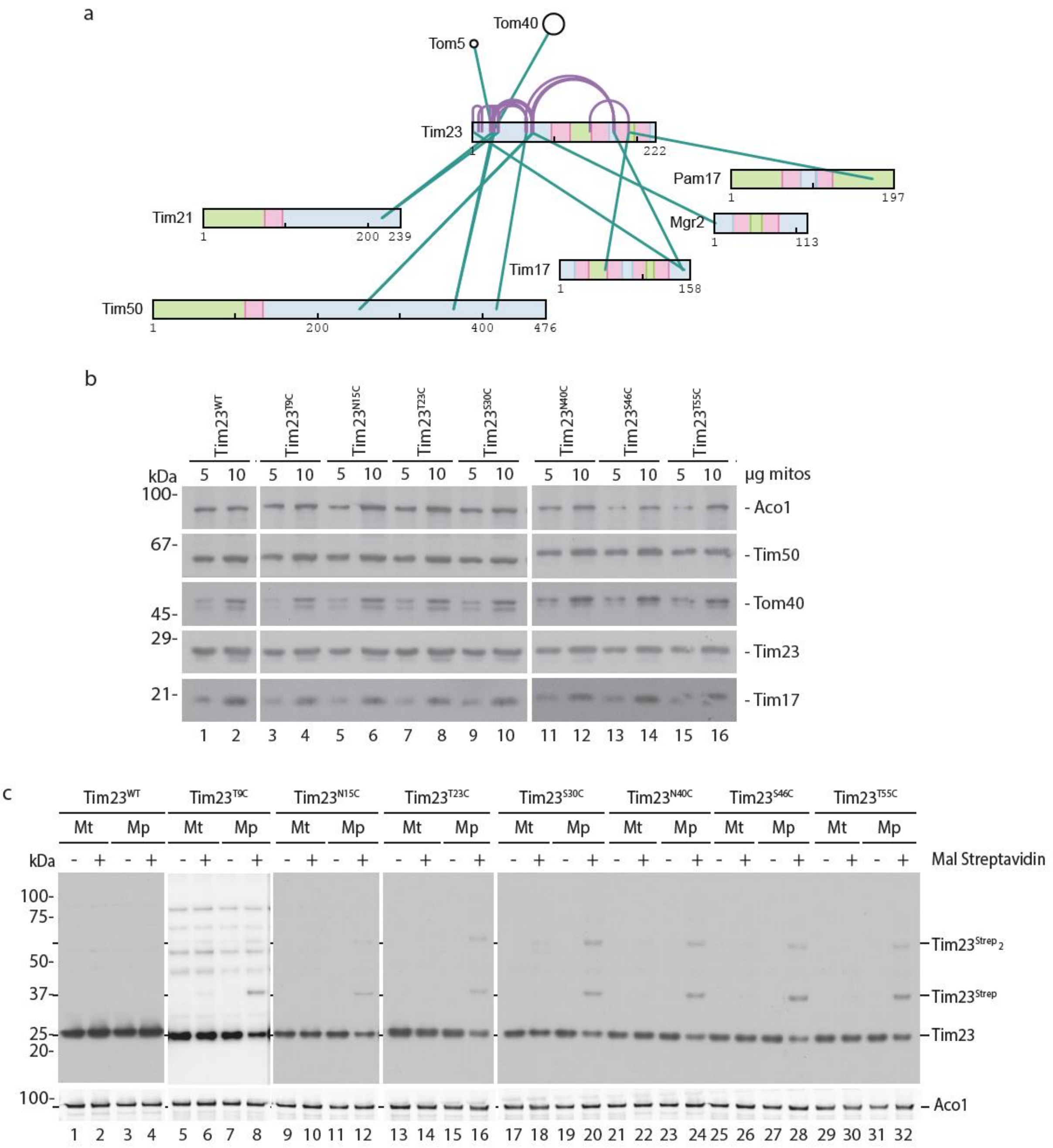

Supplementary Fig. 3. Tim23 N-terminus localizes to the IMS. a Overview of intra- and inter- protein cross-links obtained for Tim23. Colour code indicates domain location. blue: IMS, pink: IMM and green: matrix. **b** Purified mitochondria from wild type cells and cells expressing Tim23 cysteine mutants were subjected to SDS-PAGE and immunoblotting. Representative image from two independent experiments. **c** Cysteine modification assay for Tim23 was carried out in mitochondria and mitoplasts from wild type and Tim23 cysteine mutants in the presence or absence of maleimide streptavidin. Samples were subjected to SDS-PAGE and western blotting. Representative image from three replicates.

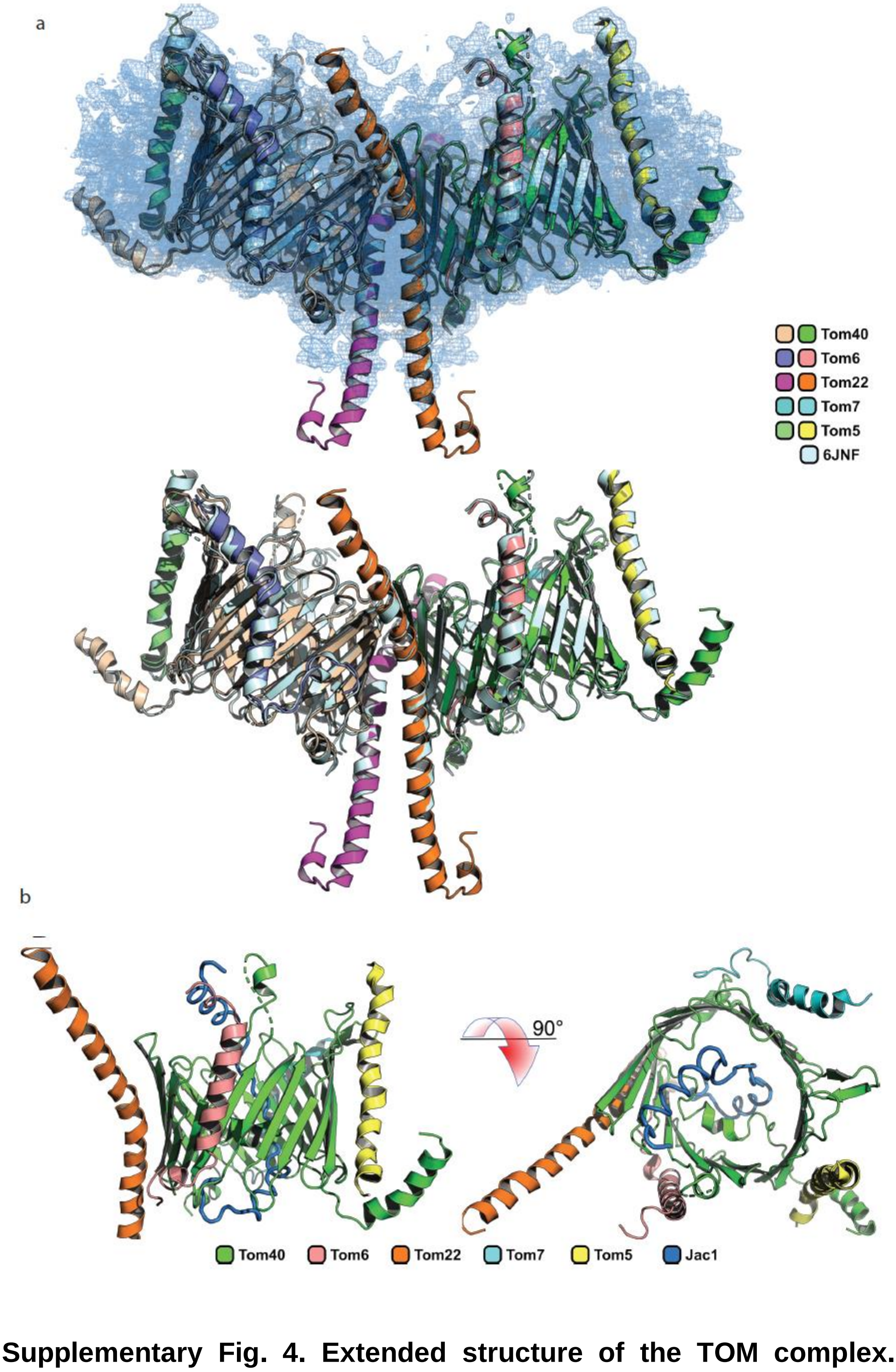

Supplementary Fig. 4. Extended structure of the TOM complex. a *de novo* extension of intermembrane space (IMS) domain of Tom22 (magenta and orange) based on available TOM complex structures (PDB id: 6JNF, EMDB ID: 9851). Rosetta tools were utilized for extending the structure. Superposition of the published TOM complex atomic model (PDB id: 6JNF) supports the extended **(**modelled) TOM complex. **b** Single TOM complex subunit with front and top view. Colour code indicates individual subunits.

Supplementary Table 1: List of identical inter-protein cross-links obtained by three methods: 1) TIM23 complex isolation followed by cross-linking, 2) whole mitochondria *in organello* cross-linking, and 3) *in organello* cross-linking followed by TIM23 complex isolation, in resting (- $Jac1^{sfGFP}$) or active (+ $Jac1^{sfGFP}$) state.

**Cross-linker**	**Inter-protein cross-links**	**Approach 1 Isolation followed by cross-linking**		**Approach 2 *in organello* cross-linking**		**Approach 3 *in organello* cross-linking followed by isolation**	
		- $Jac1^{sfGFP}$	+ $Jac1^{sfGFP}$	- $Jac1^{sfGFP}$	+ $Jac1^{sfGFP}$	- $Jac1^{sfGFP}$	+ $Jac1^{sfGFP}$
EDC	$Tom70^{103}$ - $Tom20^{183}$	-	+	-	+	NA	NA
EDC	$Tom5^{1}$ - $Tom20^{90}$	-	+	-	+	NA	NA
EDC	$Tom5^{1}$ - $Tom20^{182}$	-	+	+	-	NA	NA
EDC	$Tom5^{1}$ - $Tom20^{183}$	-	+	+	-	NA	NA
DSS	$Tim23^{190}$ - $Tim17^{55}$	+	-	-	-	+	-
DSS	$Hsp70^{445}$ - $Mge1^{104}$	+	+	+	+	+	+
DSS	$Hsp70^{83}$ - $Mge1^{119}$	-	+	-	+	+	+
DSS	$Hsp70^{444}$ - $Tim44^{228}$	-	+	-	-	-	+
DSS	$Hsp70^{514}$ - $Tim44^{215}$	+	-	-	-	+	-
DSS	$Hsp70^{574}$ - $Tim44^{198}$	+	+	-	-	+	+
DSS	$Pam16^{34}$ - $Pam18^{101}$	+	+	+	-	+	+
DSS	$Pam16^{89}$ - $Pam18^{135}$	-	+	-	-	-	+

-: cross-link not detected

+: cross-link detected

NA: not analyzed

Supplementary Table 2: List of yeast strains generated in this study.

**Strain**	**Genotype**
^{HisS*}Tim23 *tim21*Δ	^{HisS*}Tim23 *tim21::TRP*
BY4741-pRG13	BY4741 + [pRG13-Jac1^{sfGFP} (LEU2)]
Tim21^{FLAG}	YPH499 *tim21::*TIM21^{FLAG} (HIS3)
Tim21^{FLAG}+Tim21^{WT}	YPH499 *tim21::*TIM21^{FLAG} (HIS3) + [pFL39-TIM21 (TRP)]
YPH499-pRG26	YPH499 + [pRG26 (TRP)]
YPH499-pRG27 (Tim23^{ALFA})	YPH499 + [pRG27-pTIM23^{ALFA} (TRP)]
YPH499-pRG28 (Tim17^{ALFA})	YPH499 + [pRG28-pTIM17^{ALFA} (TRP)]
YPH499-pRG30 (Tim50^{ALFA})	YPH499 + [pRG30-pTIM50^{ALFA} (TRP)]
YPH499-pRG33 (Tim44^{ALFA})	YPH499 + [pRG33-pTIM44^{ALFA} (TRP)]
YPH499-pRG35 (Pam18^{ALFA})	YPH499 + [pRG35-pPAM18^{ALFA} (TRP)]
YPH499-pRG37 (Pam16^{ALFA})	YPH499 + [pRG37-pPAM16^{ALFA} (TRP)]
YPH499-R673 (Tim23^{WT})	MB29 *tim23*Δ + [pRS413-pORFt TIM23 (HIS3)]
YPH499-pRG39 (Tim23^{T9C})	MB29 *tim23*Δ + [pRS413-pORFt TIM23 T9C (HIS3)]
YPH499-pRG40 (Tim23^{N15C})	MB29 *tim23*Δ + [pRS413-pORFt TIM23 N15C (HIS3)]
YPH499-pRG41 (Tim23^{T23C})	MB29 *tim23*Δ + [pRS413-pORFt TIM23 T23C (HIS3)]
YPH499-pRG42 (Tim23^{S30C})	MB29 *tim23*Δ + [pRS413-pORFt TIM23 S30C (HIS3)]
YPH499-pRG44 (Tim23^{N40C})	MB29 *tim23*Δ + [pRS413-pORFt TIM23 N40C (HIS3)]
YPH499-pRG45 (Tim23^{S46C})	MB29 *tim23*Δ + [pRS413-pORFt TIM23 S46C (HIS3)]
YPH499-pRG46 (Tim23^{T55C})	MB29 *tim23*Δ + [pRS413-pORFt TIM23 T55C (HIS3)]

Supplementary Table 3: List of plasmids used in this study.

**Plasmids**	**Description**
GST-nGFP-Strep (R286)	GST-Thrombin-GFP Nanobody-Strep in pGEX-4T3 (Amp)
pRG9	14HIS-SUMO-Jac1$^{sfGFP\ Q7R}$ in pSUMO (Kan)
Tim44$^{C\text{-}term}$ (R662)	Tim44 residues 244-431 in pPROEX HTc (Amp)
Tim44 (1014)	Tim44 full length (without presequence) in pQE60 (Amp)
pRG13	Jac1$^{sfGFP\ Q7R}$ in p425Gal1 (Amp, LEU2)
pRG26	ALFA-ALDH terminator in pRS414 (Amp, TRP1)
pRG27	Tim23 in pRG26 (Amp, TRP1)
pRG28	Tim17 in pRG26 (Amp, TRP1)
pRG30	Tim50 in pRG26 (Amp, TRP1)
pRG33	Tim44 in pRG26 (Amp, TRP1)
pRG35	Pam18 in pRG26 (Amp, TRP1)
pRG37	Pam16 in pRG26 (Amp, TRP1)
pFL39 (R84)	empty vector for yeast expression (Amp, TRP1)
pGB 9607-7 (R85)	Tim21 in pFL39 for expression in yeast (Amp, TRP1)
R673	pORFt Tim23 in pRS413 (Amp, HIS3)
pRG39	pORFt Tim23 T9C in pRS413 (Amp, HIS3)
pRG40	pORFt Tim23 N15C in pRS413 (Amp, HIS3)
pRG41	pORFt Tim23 T23C in pRS413 (Amp, HIS3)
pRG42	pORFt Tim23 S30C in pRS413 (Amp, HIS3)
pRG44	pORFt Tim23 N40C in pRS413 (Amp, HIS3)
pRG45	pORFt Tim23 S46C in pRS413 (Amp, HIS3)
pRG46	pORFt Tim23 T55C in pRS413 (Amp, HIS3)

Supplementary Table 4: Antibodies used in this study.

Antibody	Source	Identifier	Dilution
GFP (western blot)	Roche	11814460001	1:1000
GFP (microscopy)	Thermo Fisher Scientific	A-11120	
Anti-ALFA HRP-conjugated	NanoTag Biotechnologies	N1502-HRP	1:500
FLAG	Sigma	F3165	1:500
Peroxidase Streptavidin (SA-HRP)	Jackson ImmunoResearch Lab	016-030-084	1:500
Goat anti Rabbit IgG (H+L) HRPO	Jackson ImmunoResearch Lab	111-035-144	1:10,000
Tim23	Rabbit polyclonal, self-made	3846	1:500
Tim23^{IMS}	Rabbit polyclonal, self-made	133	1:750
Tim17	Rabbit polyclonal, self-made	4968	1:200
Tim50	Rabbit polyclonal, self-made	3314	1:400
Tim21	Rabbit polyclonal, self-made	3111	1:400
Tim44	Rabbit polyclonal, self-made	3869	1:250
Hsp70	Rabbit polyclonal, self-made	4945	1:50,000
Pam16	Rabbit polyclonal, self-made	3872	1:200
Pam18	Rabbit polyclonal, self-made	3955	1:200
Pam17	Rabbit polyclonal, self-made	5017	1:400
Tom70	Rabbit polyclonal, self-made	3530	1:400
Tom40	Rabbit polyclonal, self-made	4901	1:200
Tom22	Rabbit polyclonal, self-made	3533	1:400
Tom20	Rabbit polyclonal, self-made	3534	1:400
Tom5	Rabbit polyclonal, self-made	162	1:500
Aco1	Rabbit polyclonal, self-made	5004	1:500
