# Supplementary material for: Mapping protein interactions in the active TOM-TIM23 supercomplex
Source: Nat Commun. 2021 Sep 29;12:5715. doi: 10.1038/s41467-021-26016-1 (PMC8481542; doi:10.1038/s41467-021-26016-1)
Supplement: Supplementary file 5 — Description of additional supplementary files [file 41467_2021_26016_MOESM5_ESM.docx]

Description of additional supplementary files

Title: Supplementary Movie 1

Description: Movie depicting components of the modelled TOM-TIM23 supercomplex and the integrated intra- and inter-protein cross-links obtained from DSS, EDC and SDA cross-linking.

Title: Supplementary Data 1

Description: Mass spectrometric analyses using three approaches. 1) TIM23 complex isolation in absence or presence of Jac1sfGFP followed by DSS, SDA and EDC cross-linking, 2) DSS and EDC cross-linking on purified mitochondria in absence or presence of Jac1sfGFP, and 3) DSS cross-linking of mitochondria in absence or presence of Jac1sfGFP followed by TIM23 complex isolation. For approach 3, Tim23 WT residues are mentioned in brackets next to the HisS*-tagged residue number.

Title: Supplementary Data 2

Description: Overview of critical inter-protein cross-links obtained from the three cross-linking approaches.
