# Supplementary material for: Mapping protein interactions in the active TOM-TIM23 supercomplex
Source: Nat Commun. 2021 Sep 29;12:5715. doi: 10.1038/s41467-021-26016-1 (PMC8481542; doi:10.1038/s41467-021-26016-1)

Figure 1c

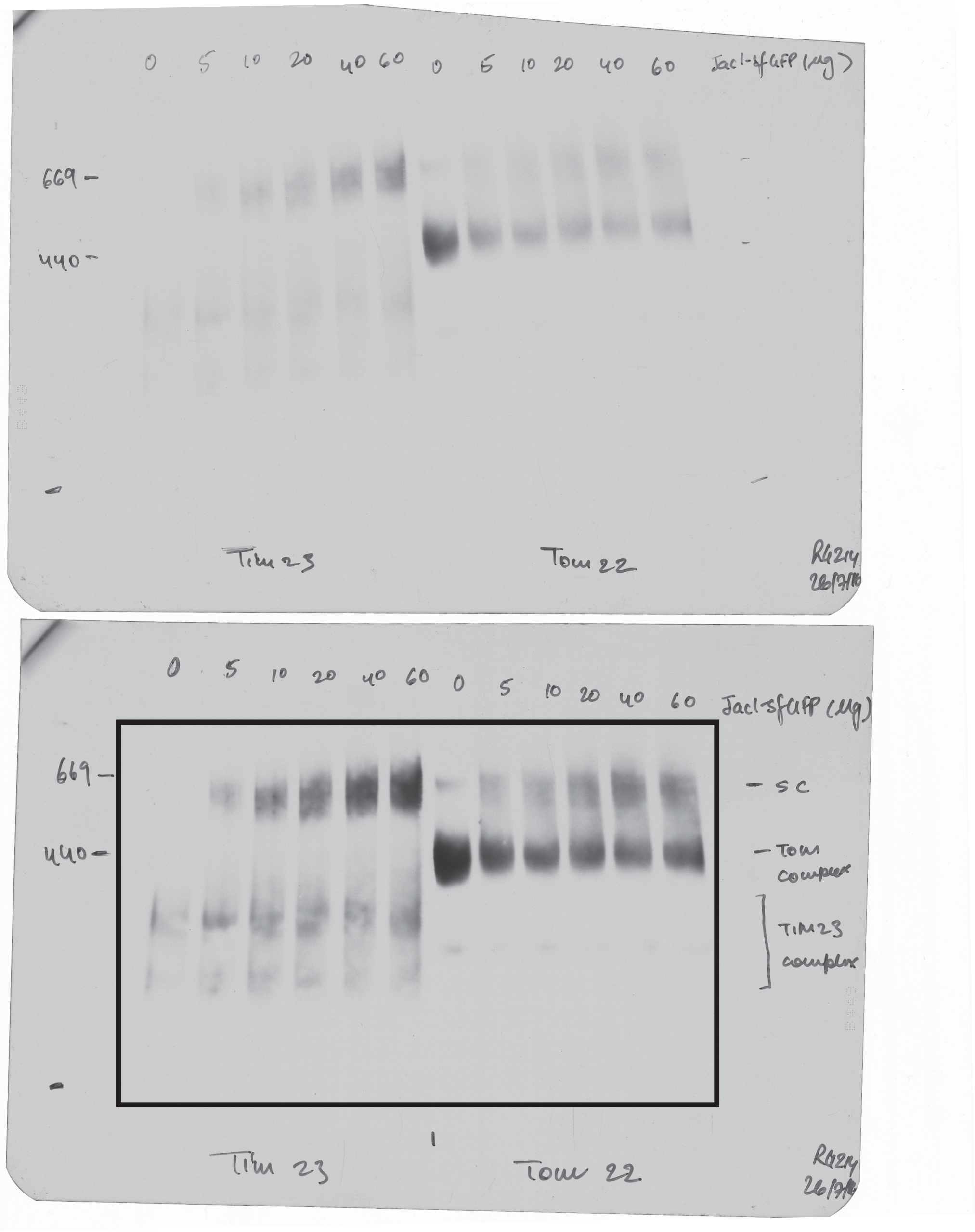

Figure 1d

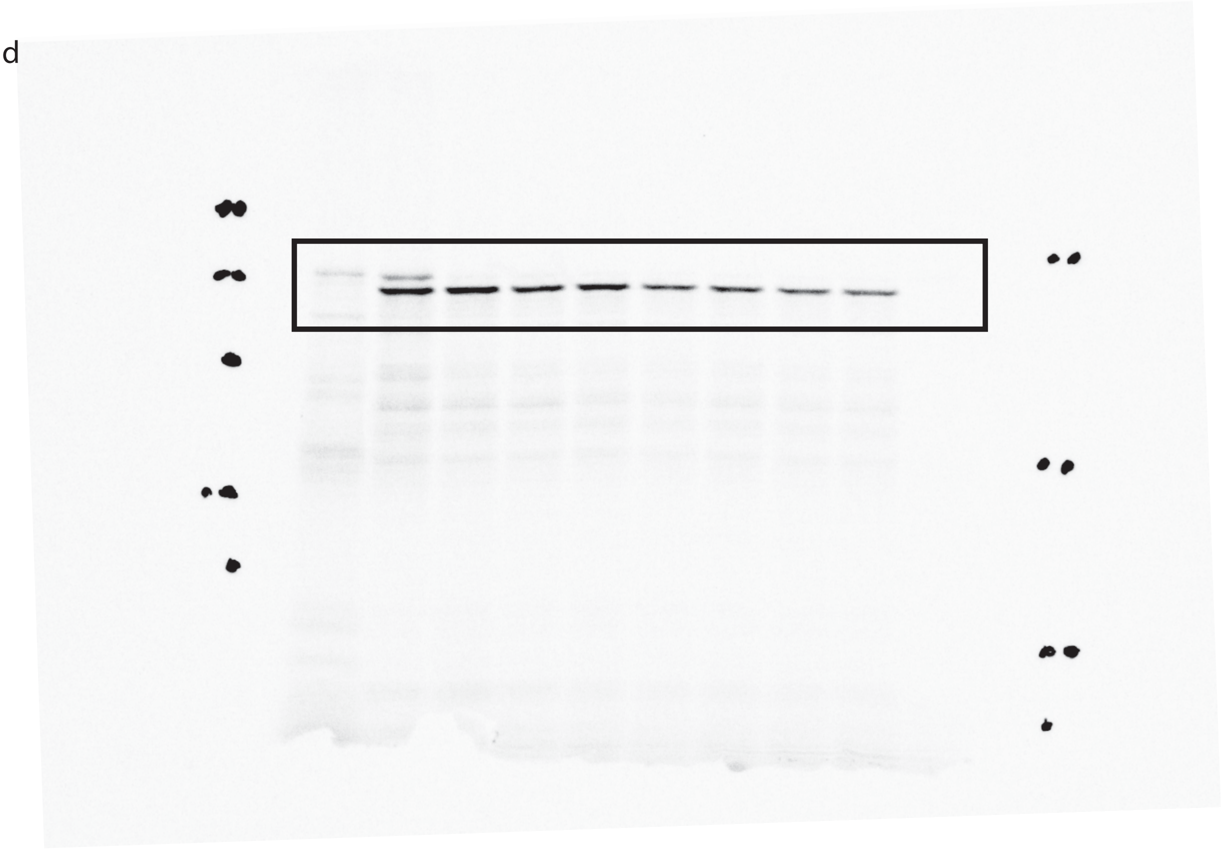

Figure 2a

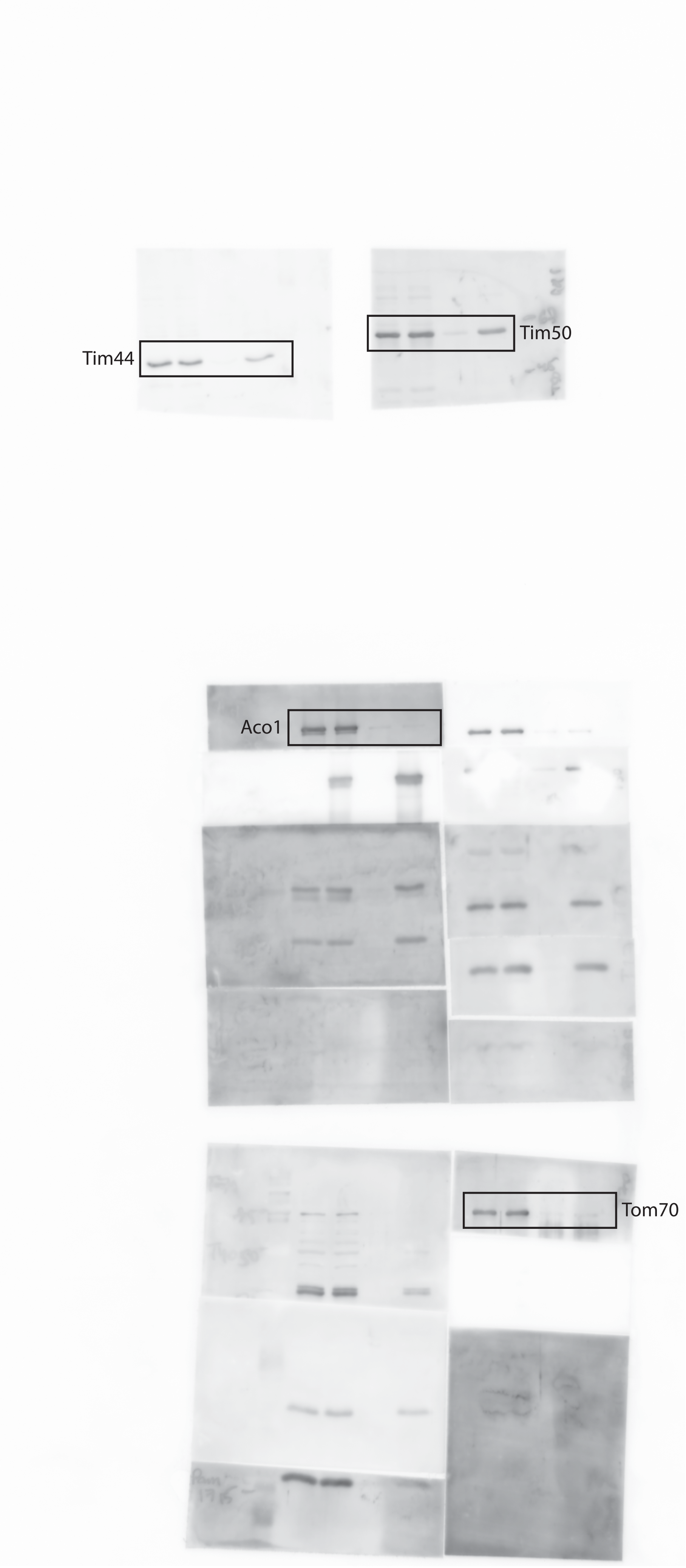

Figure 2a

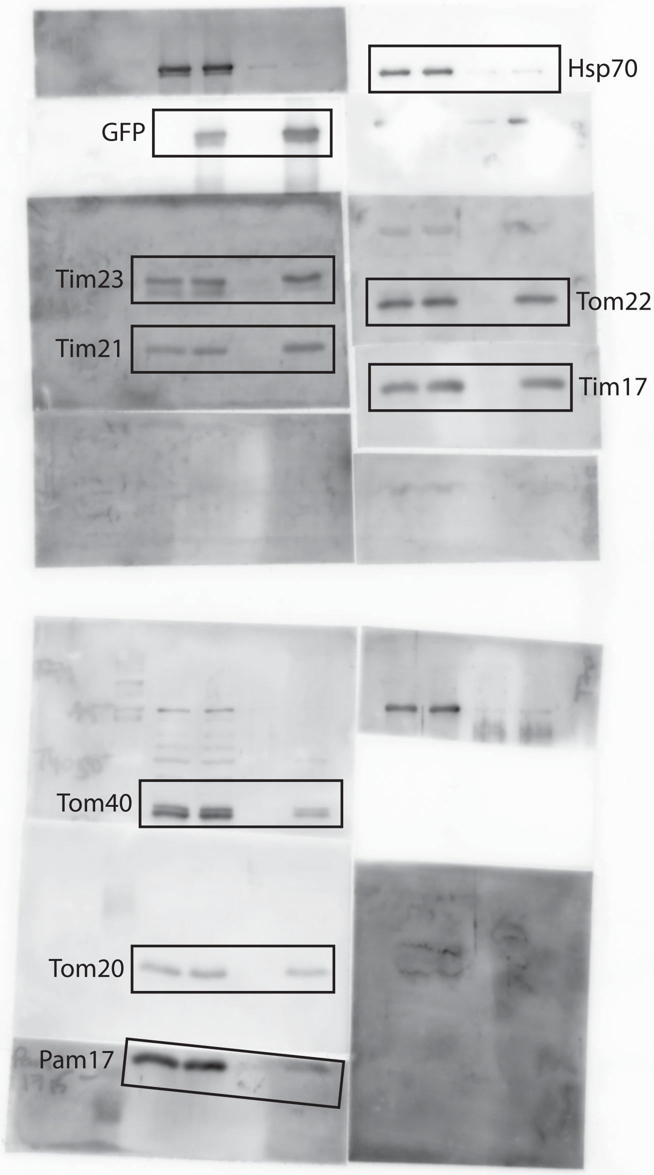

Figure 2a

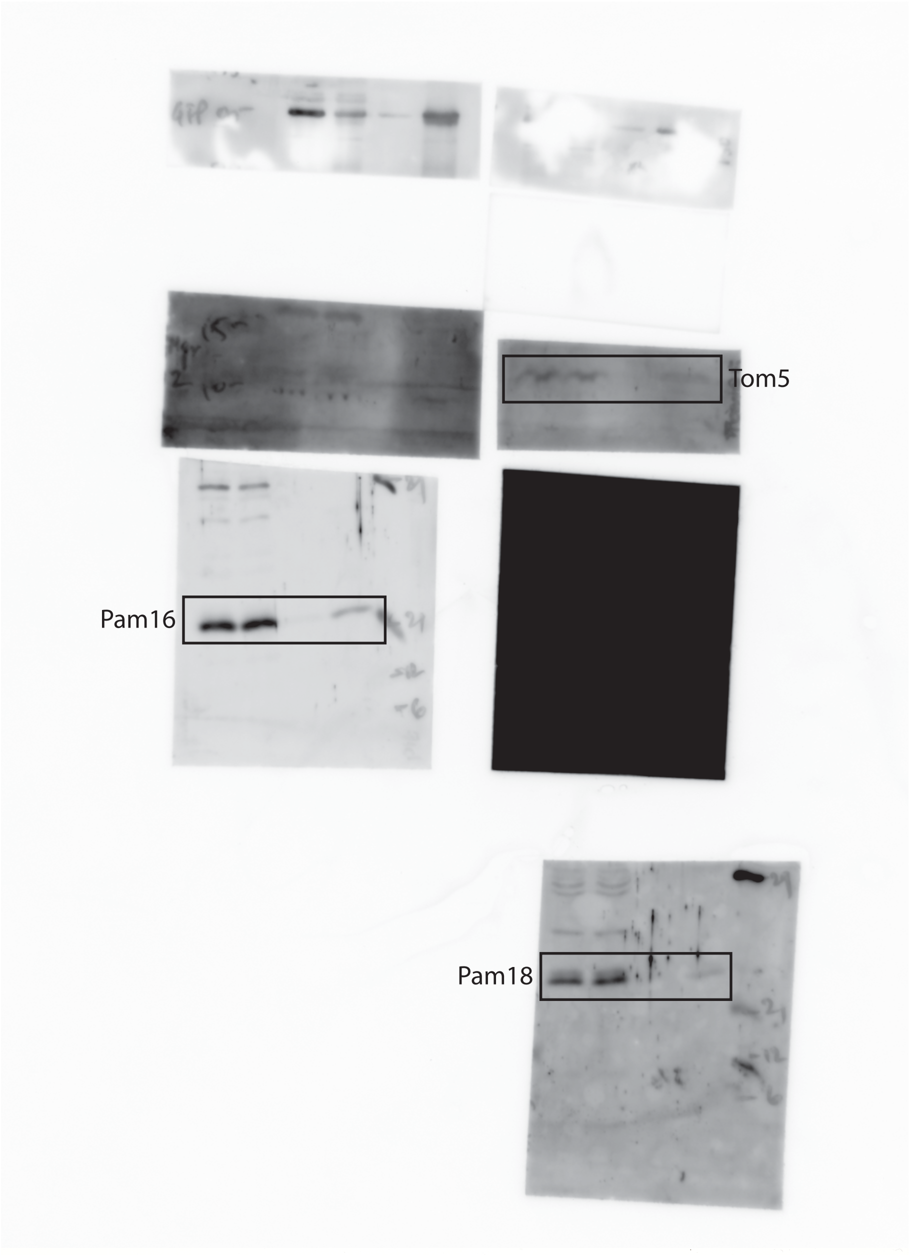

Figure 2b

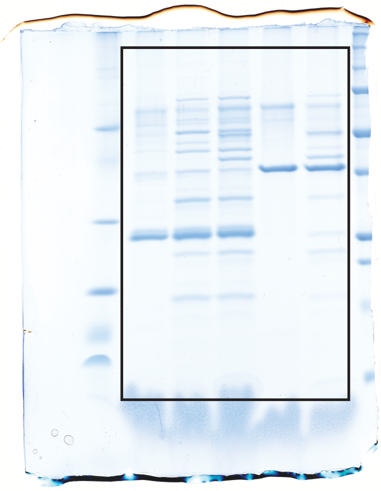

Figure 2c

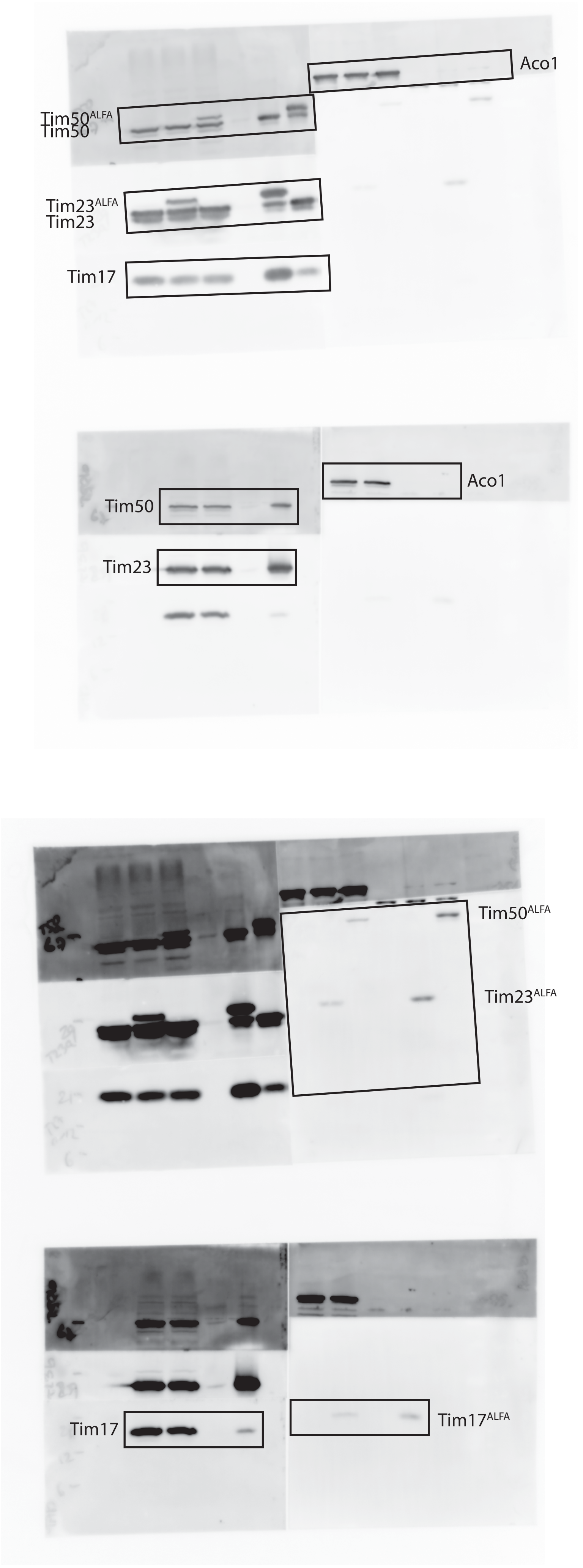
Aco1
Tim50ALFA
Tim50
Tim23ALFA
Tim23
Tim17
Aco1
Tim50
Tim23
Tim50ALFA
Tim23ALFA
Tim17
Tim17ALFA

Figure 2d

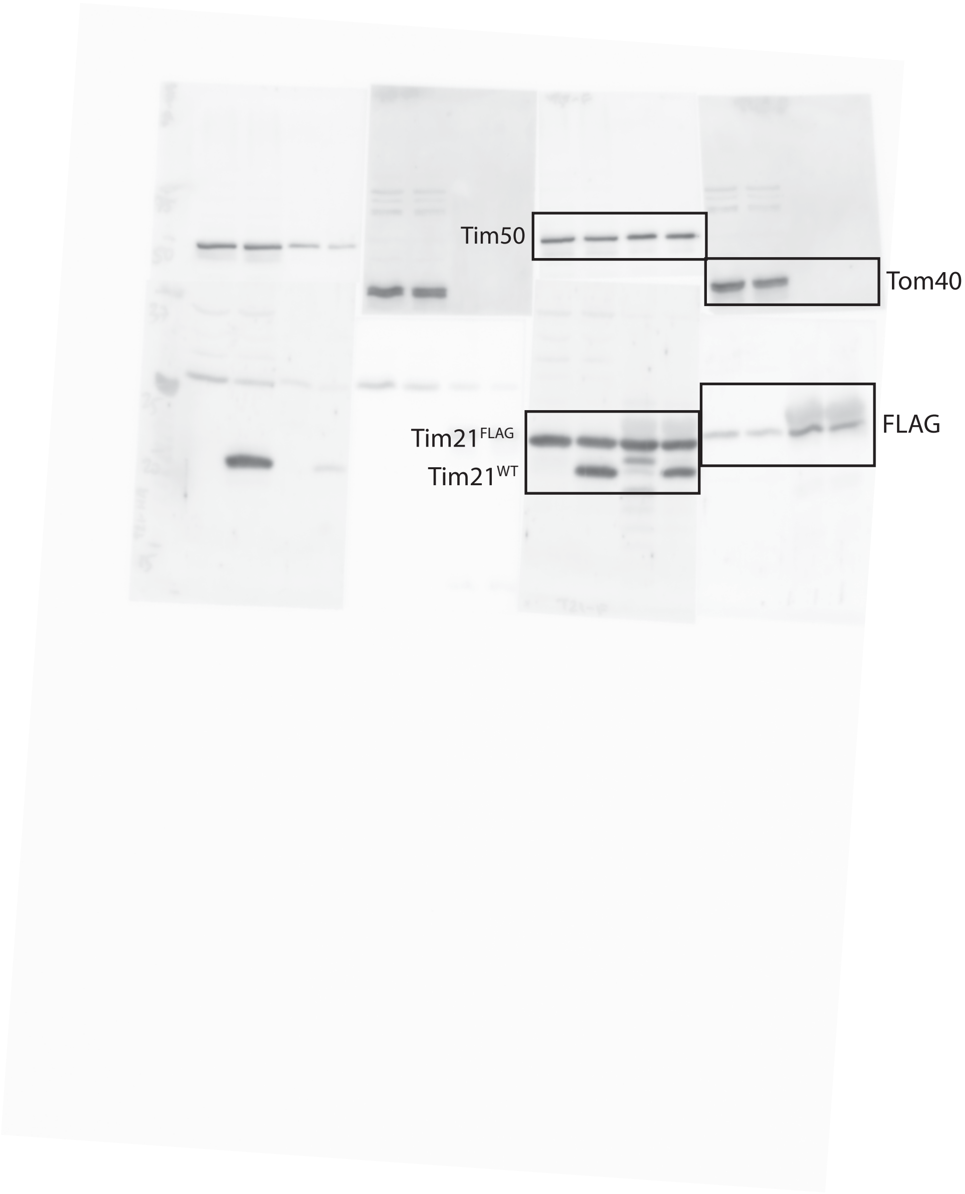

Figure 2e

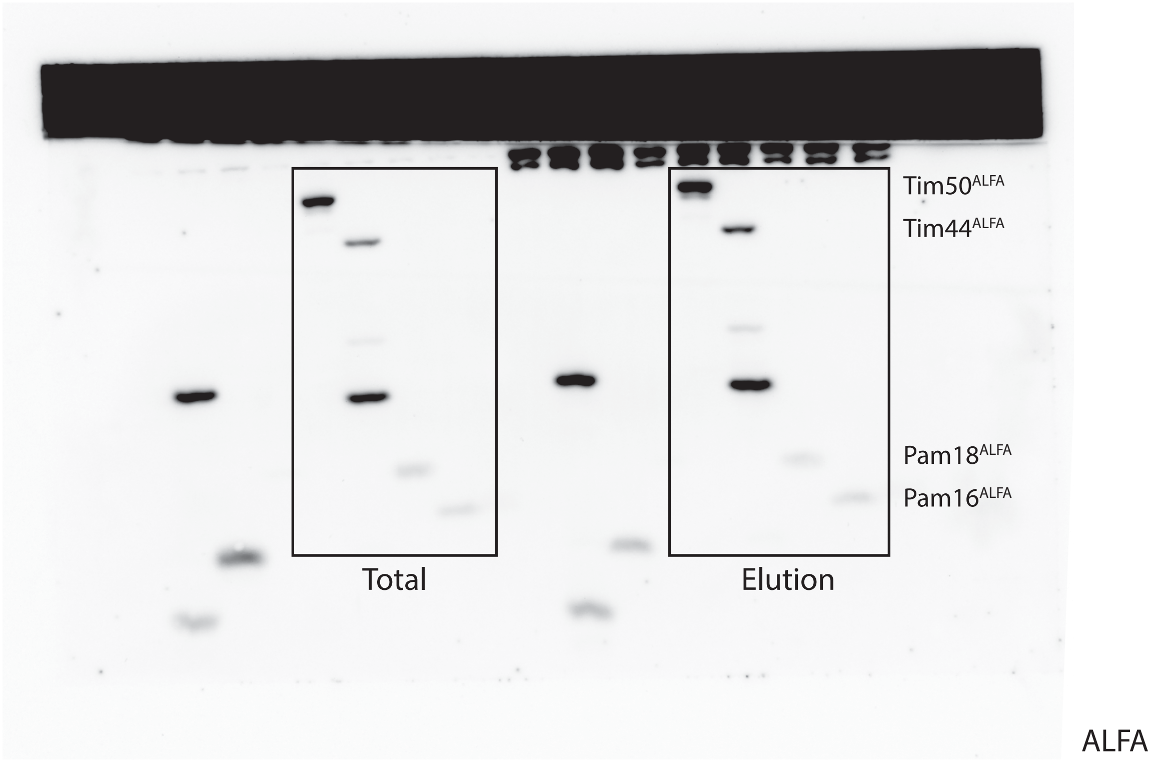

Figure 2e

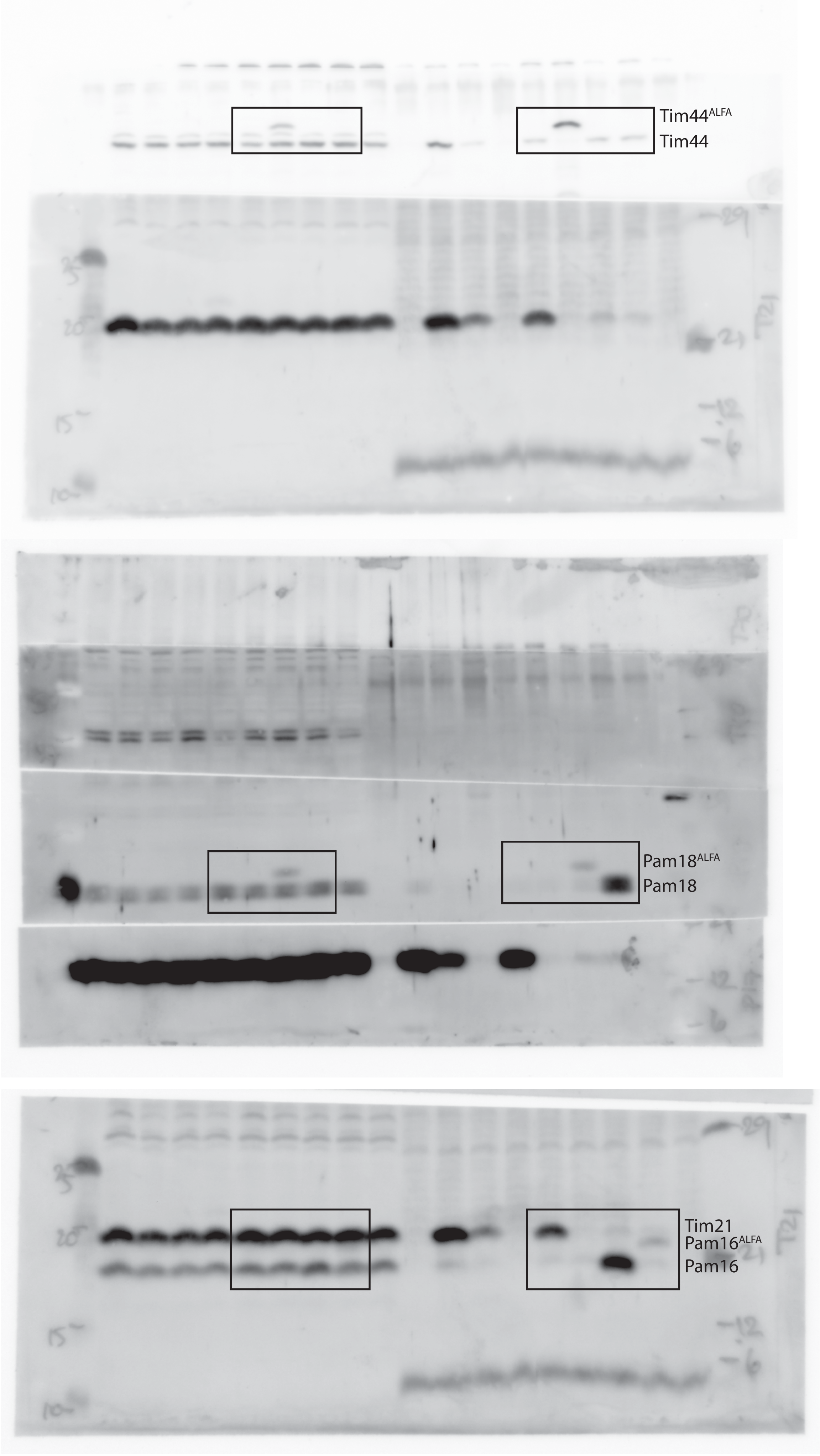

Figure 7a

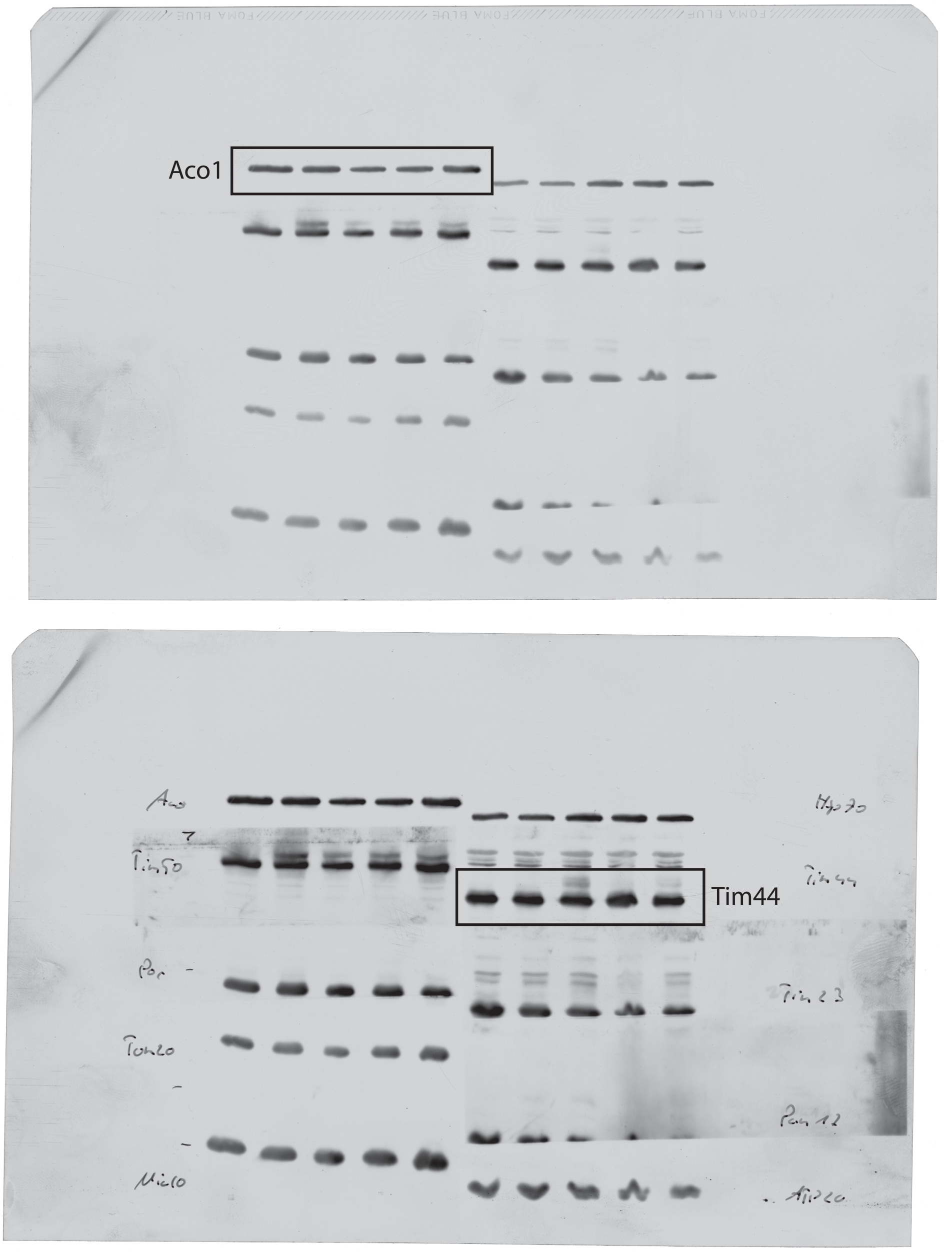

Figure 7b

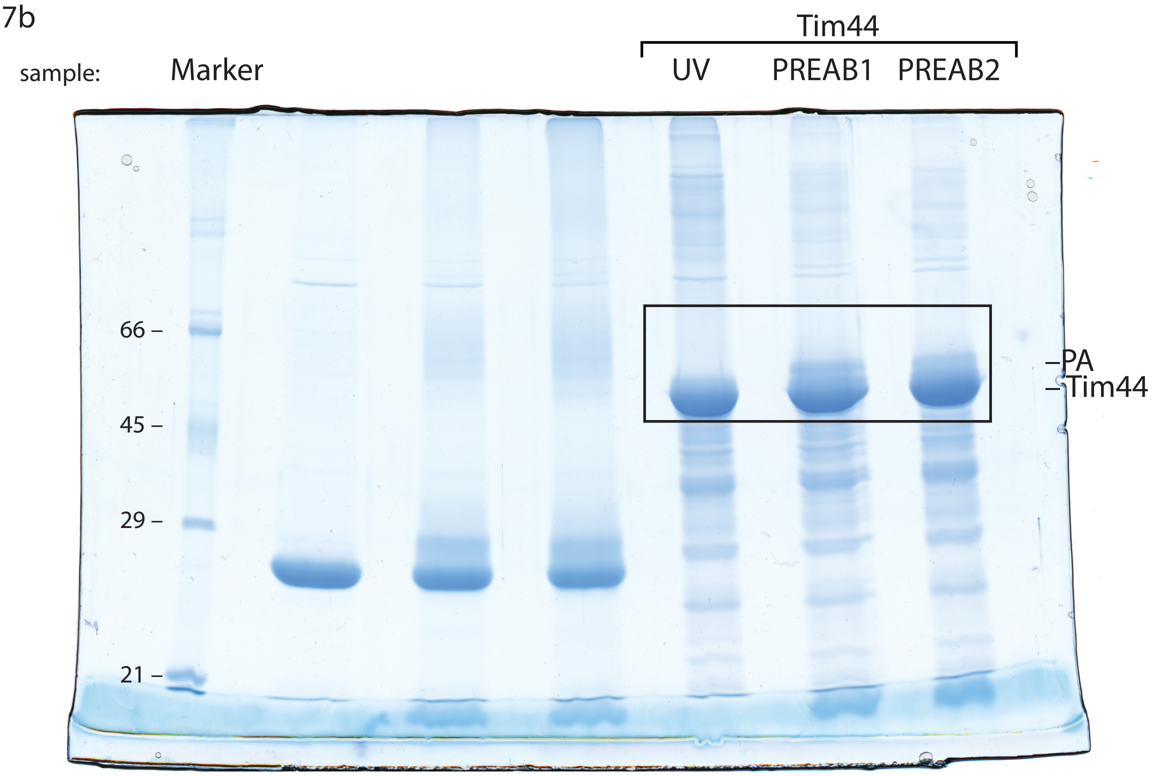

Figure 7b

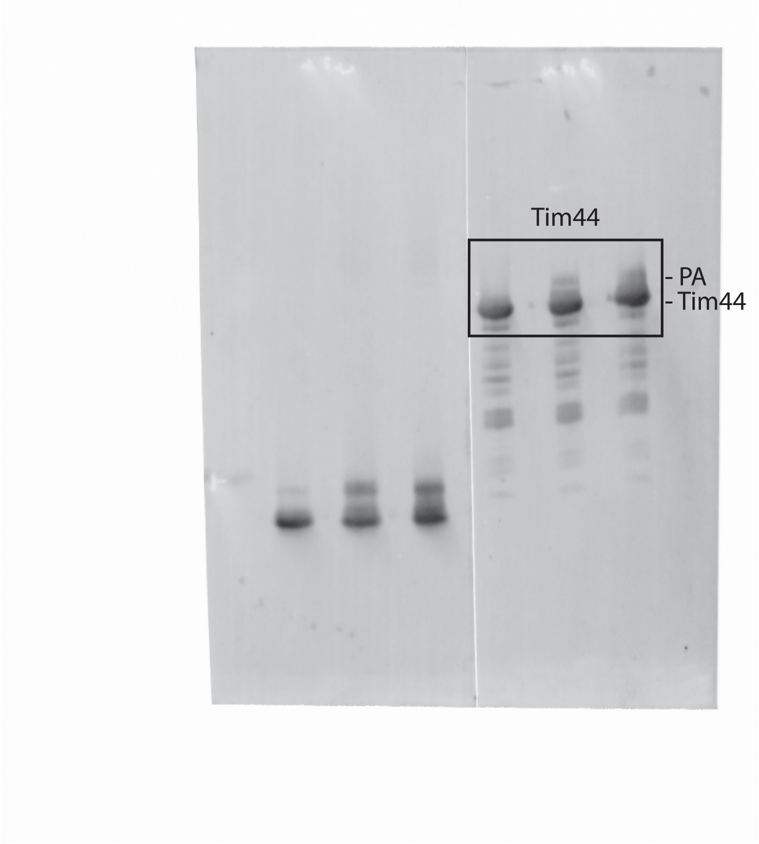

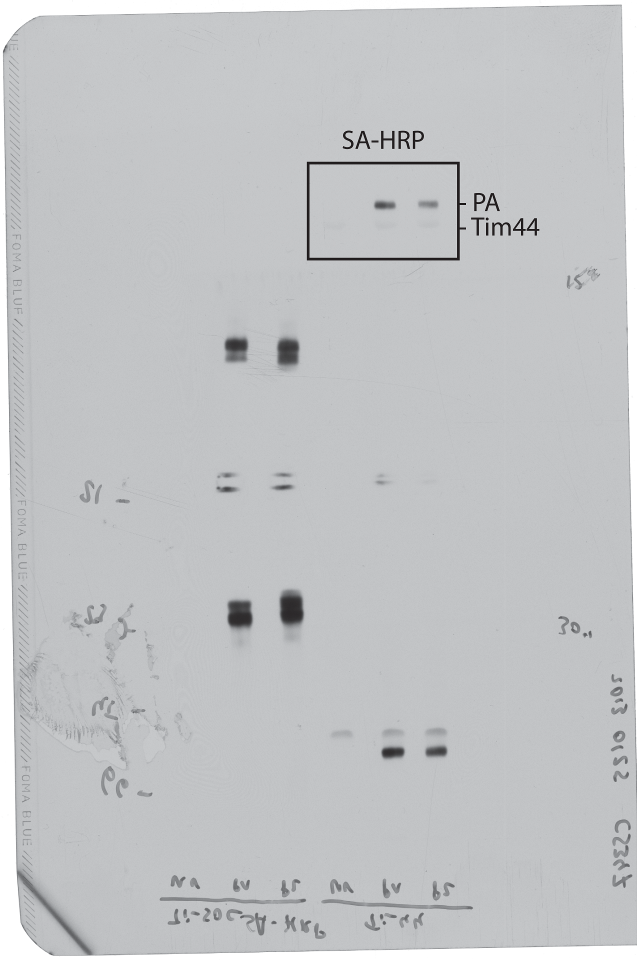

Figure 7c

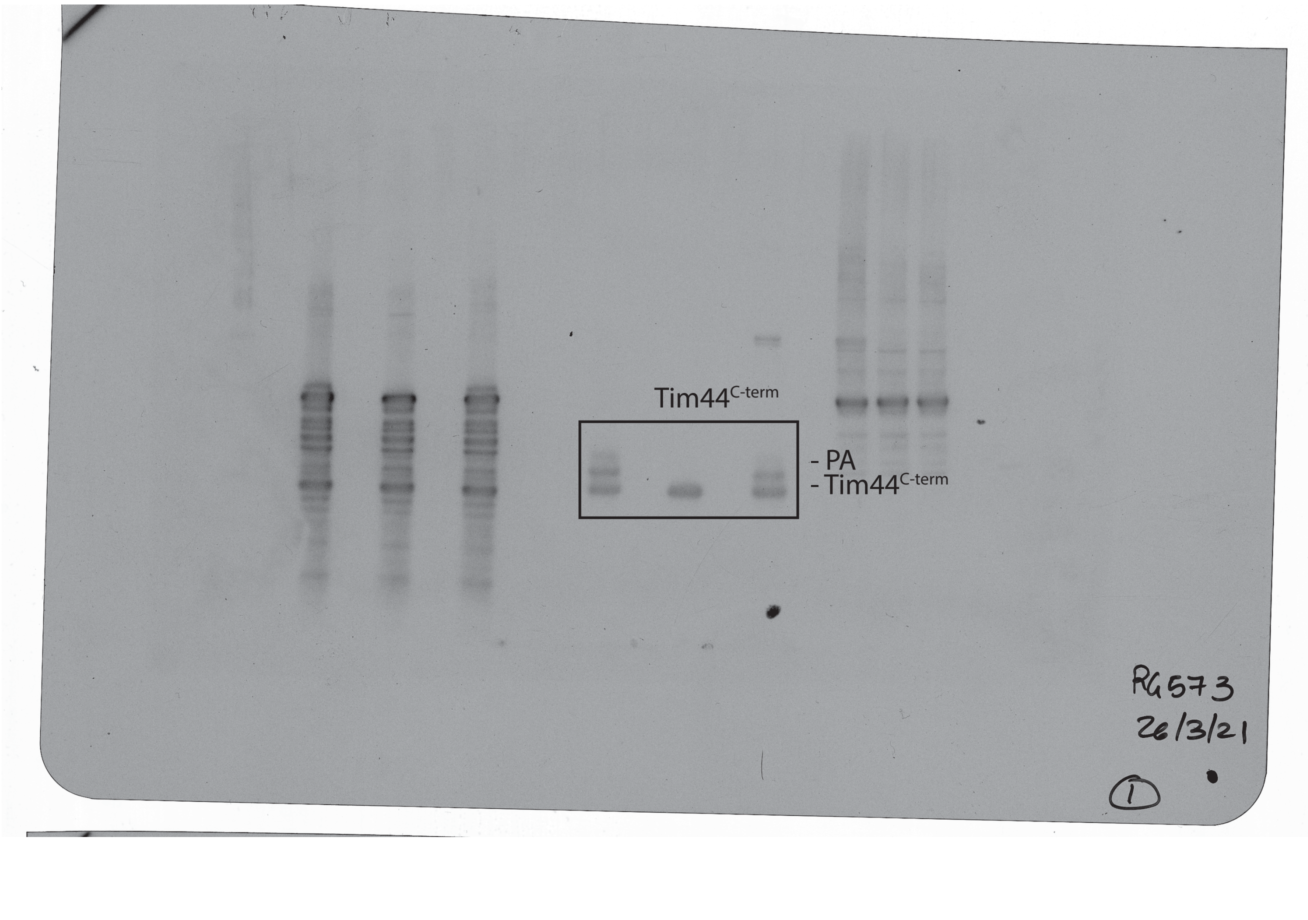

Figure 7c

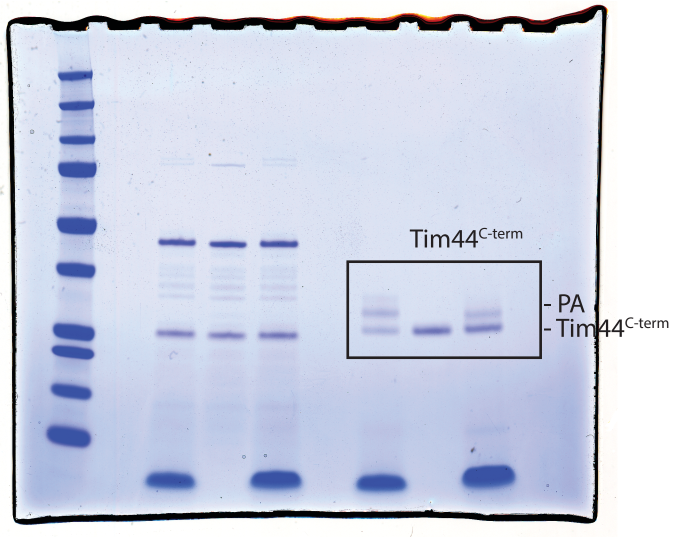

Supplementary Figure 1c

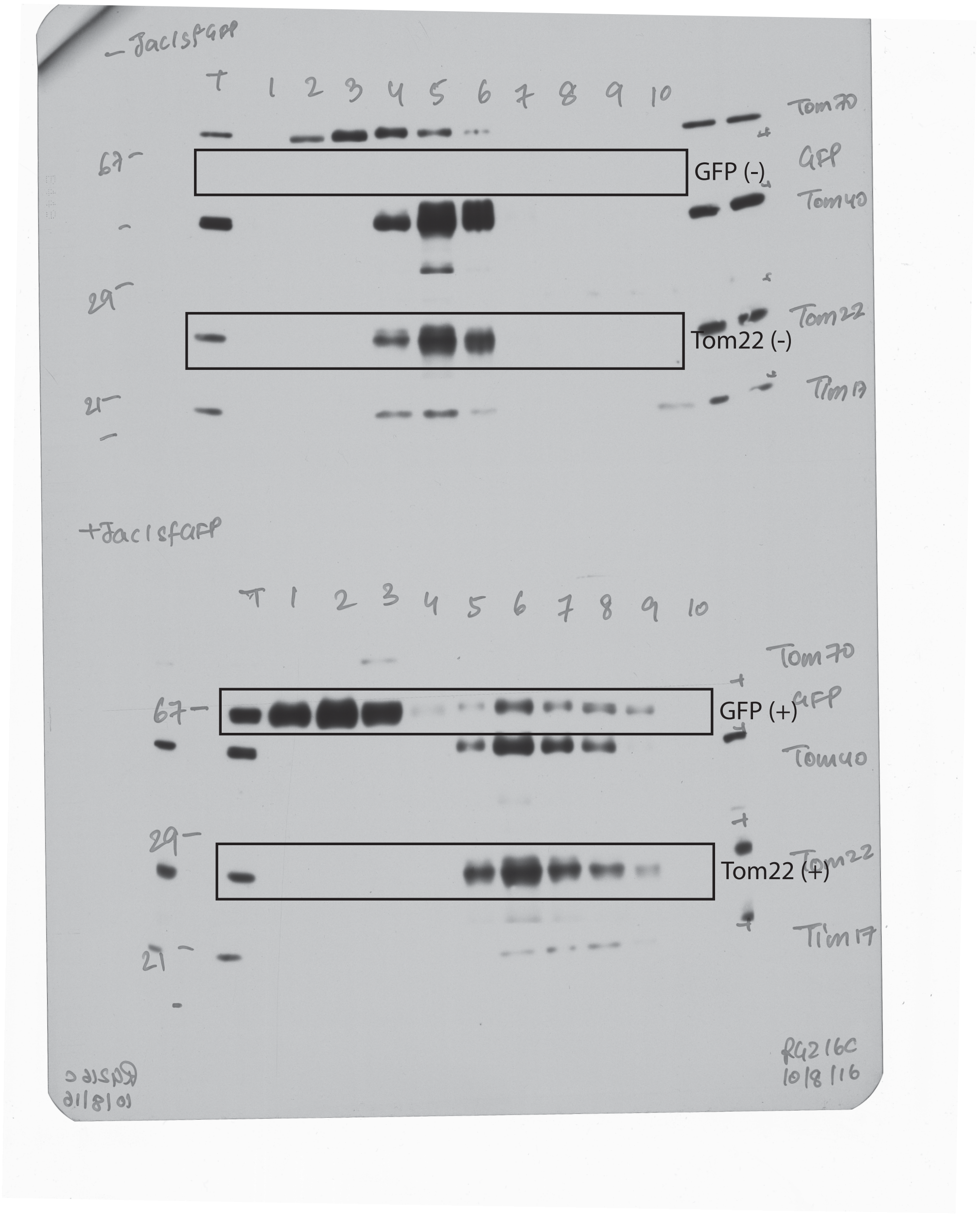

Supplementary Figure 1c

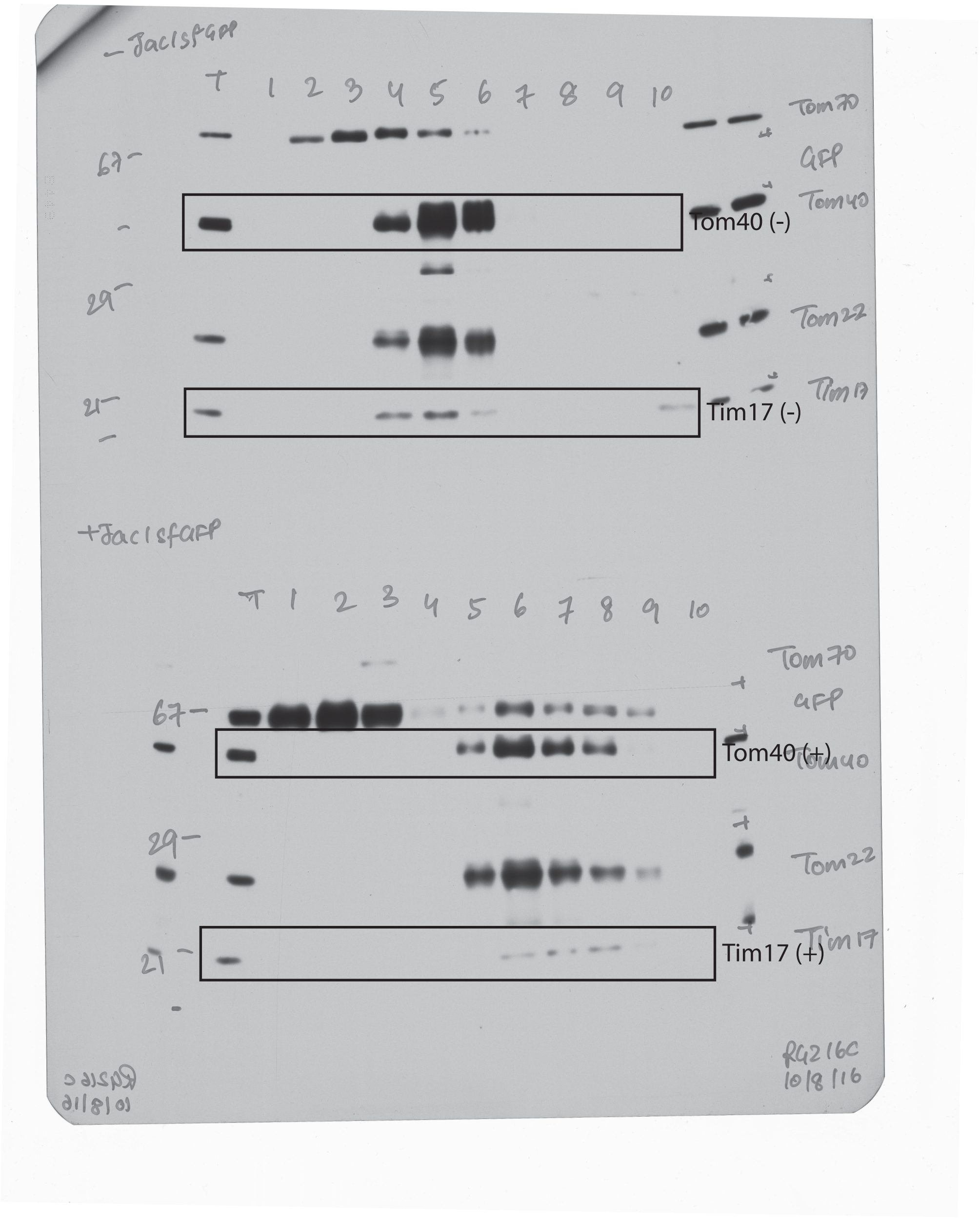

Supplementary Figure 1c

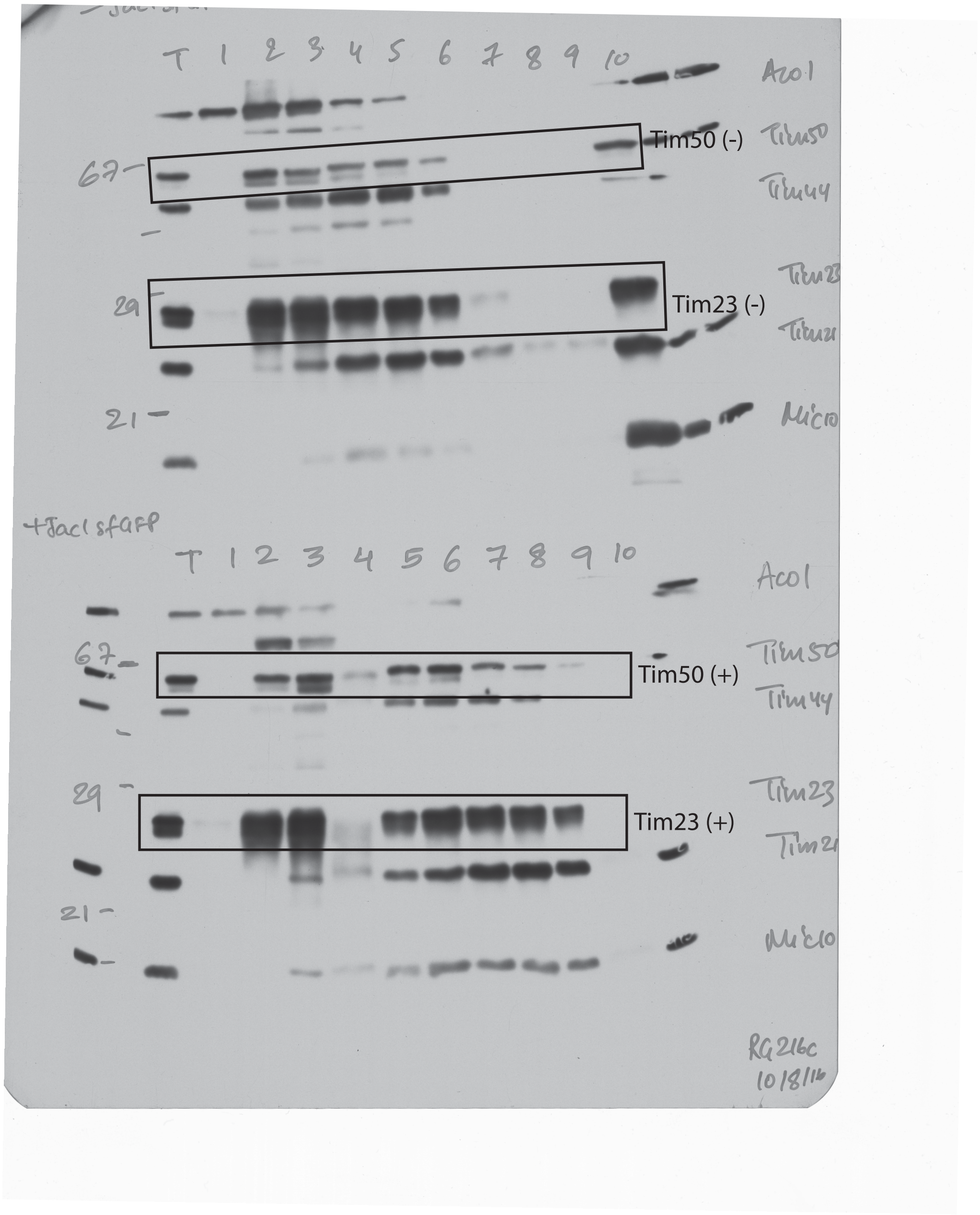

Supplementary Figure 1c

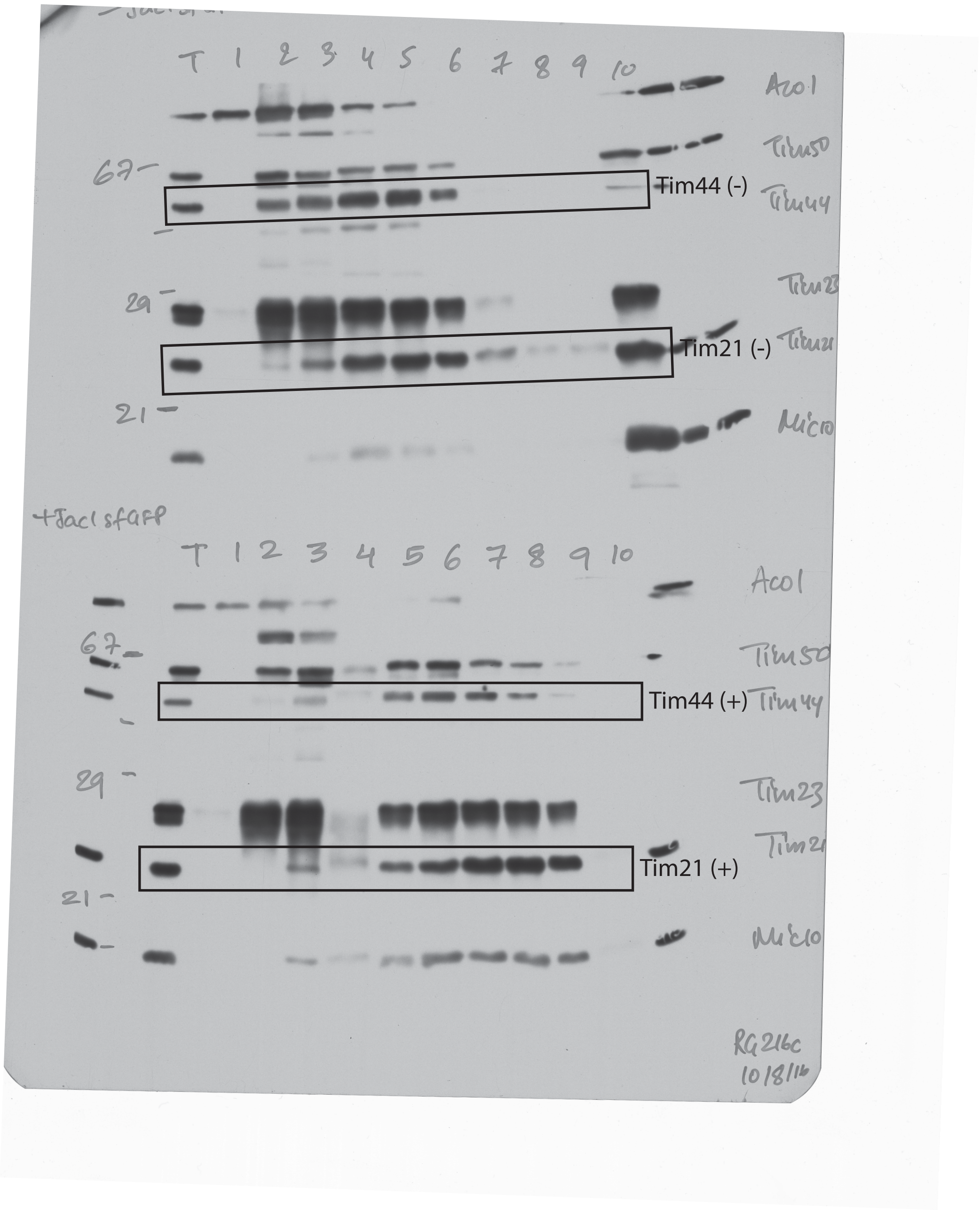

Supplementary Figure 2a

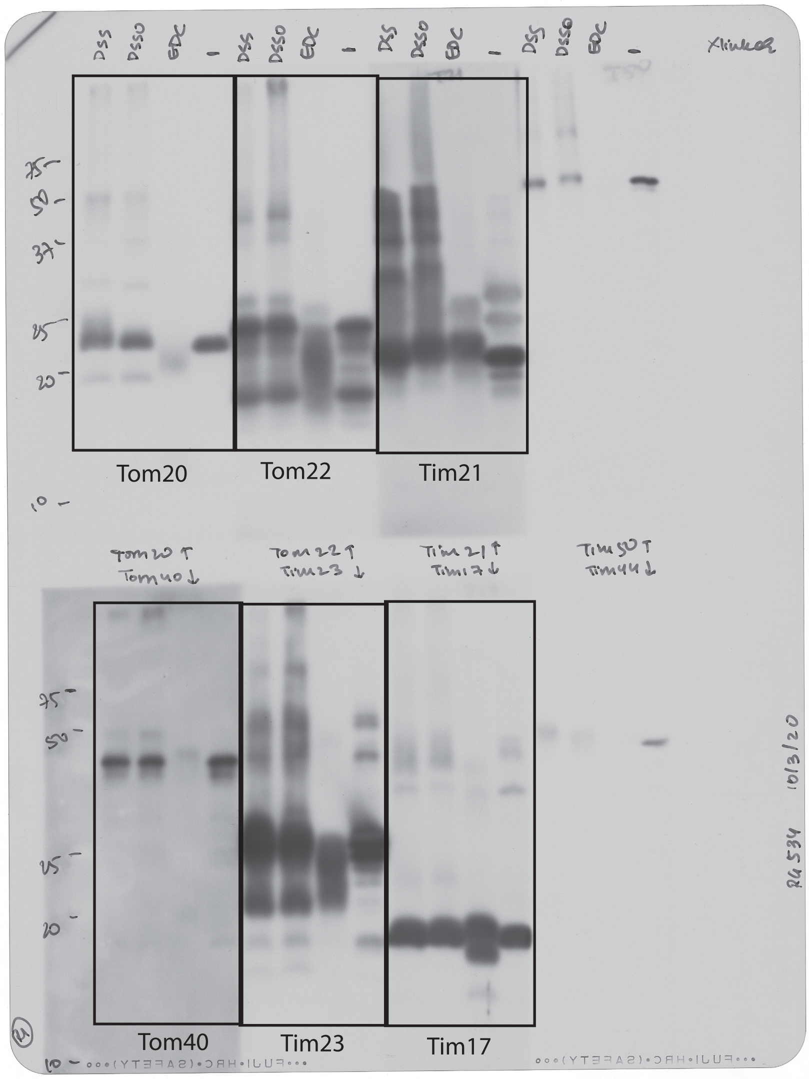

Supplementary Figure 2a

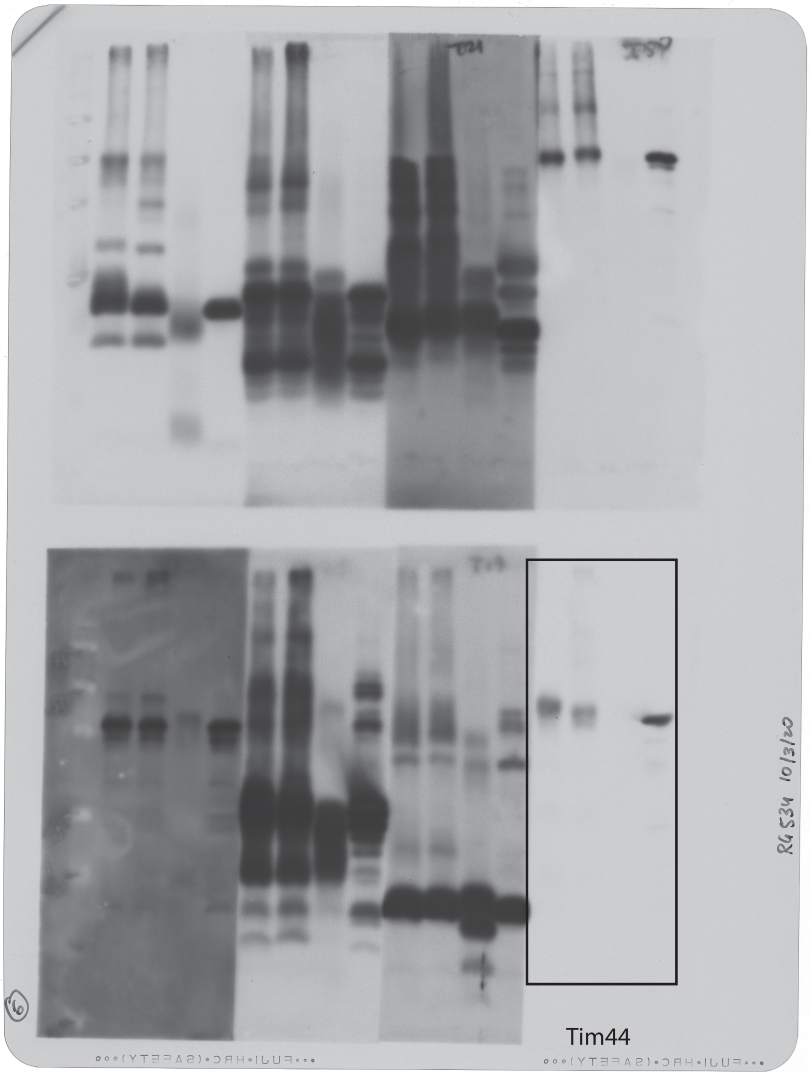

Supplementary Figure 2b

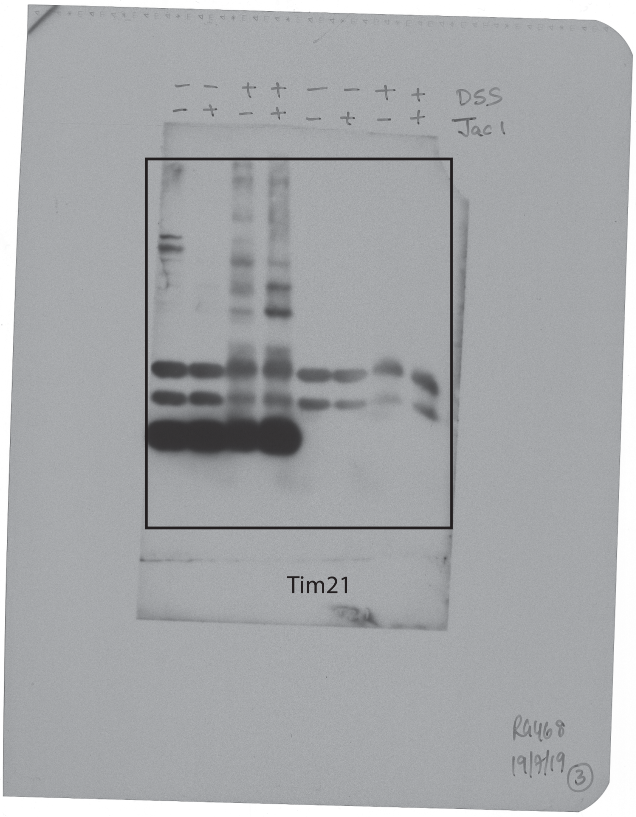

Supplementary Figure 2b

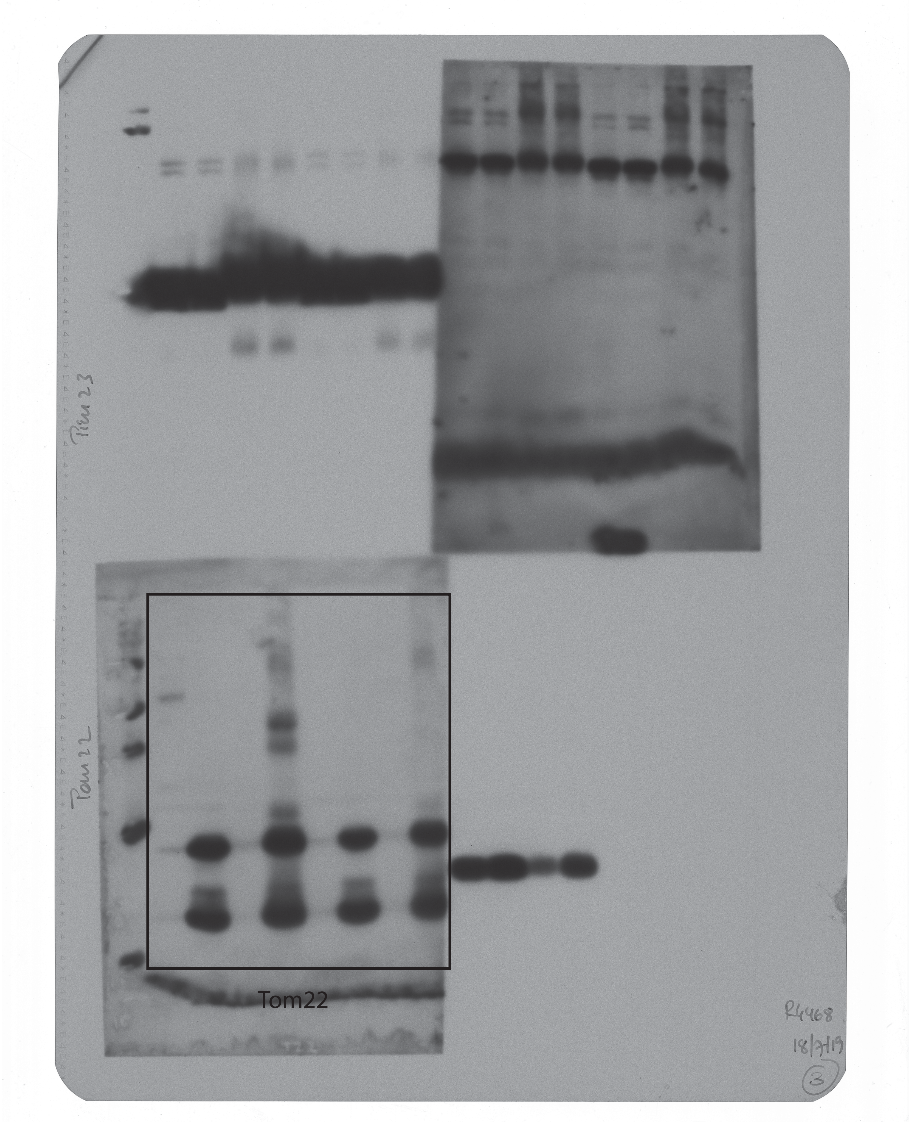

Supplementary Figure 3b

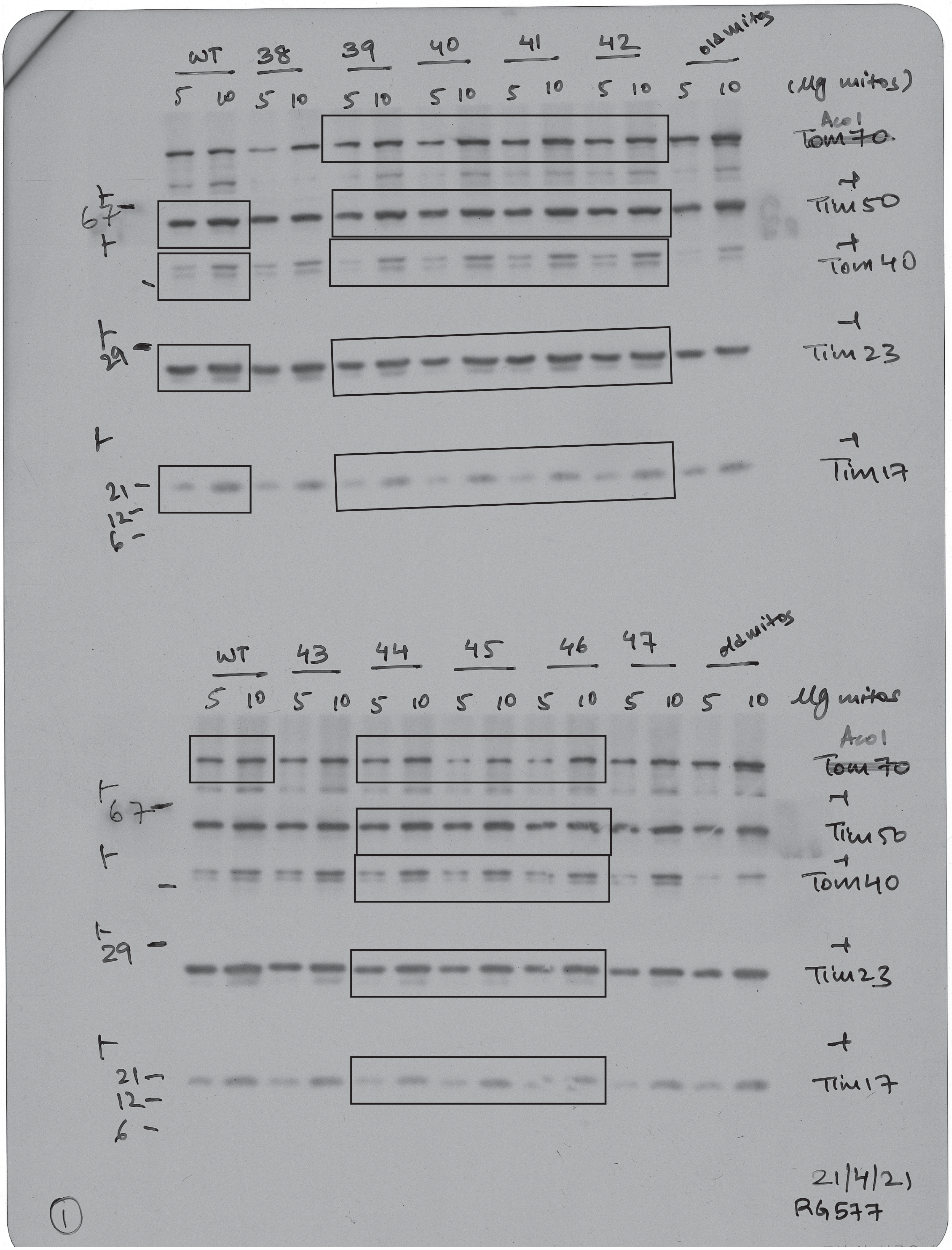

Supplementary Figure 3c (bottom panel)

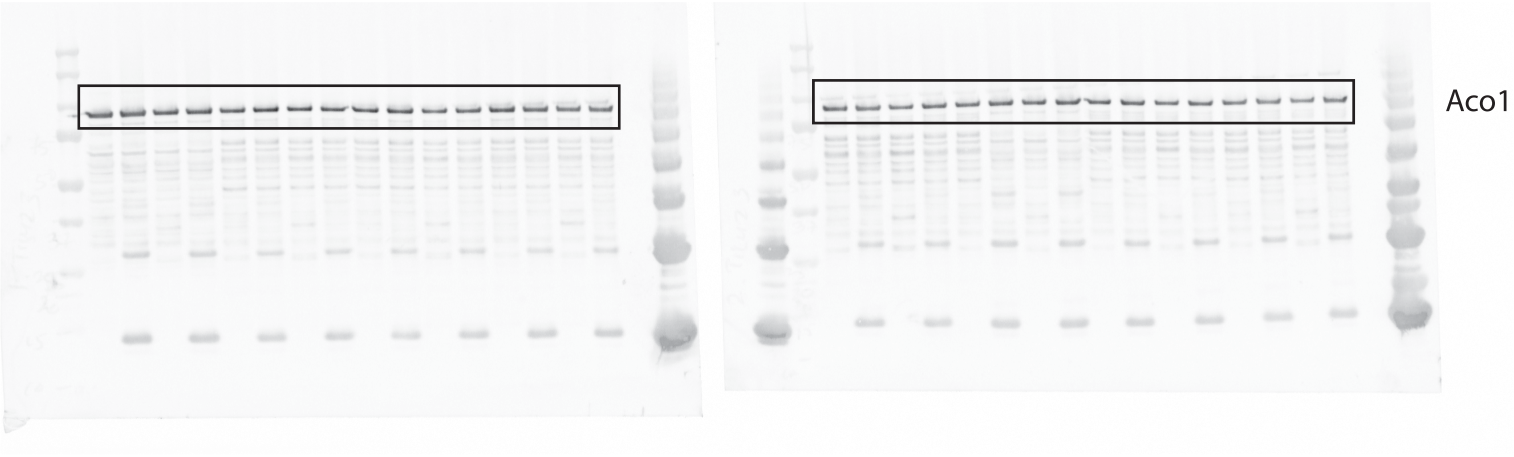

Supplementary Figure 3c

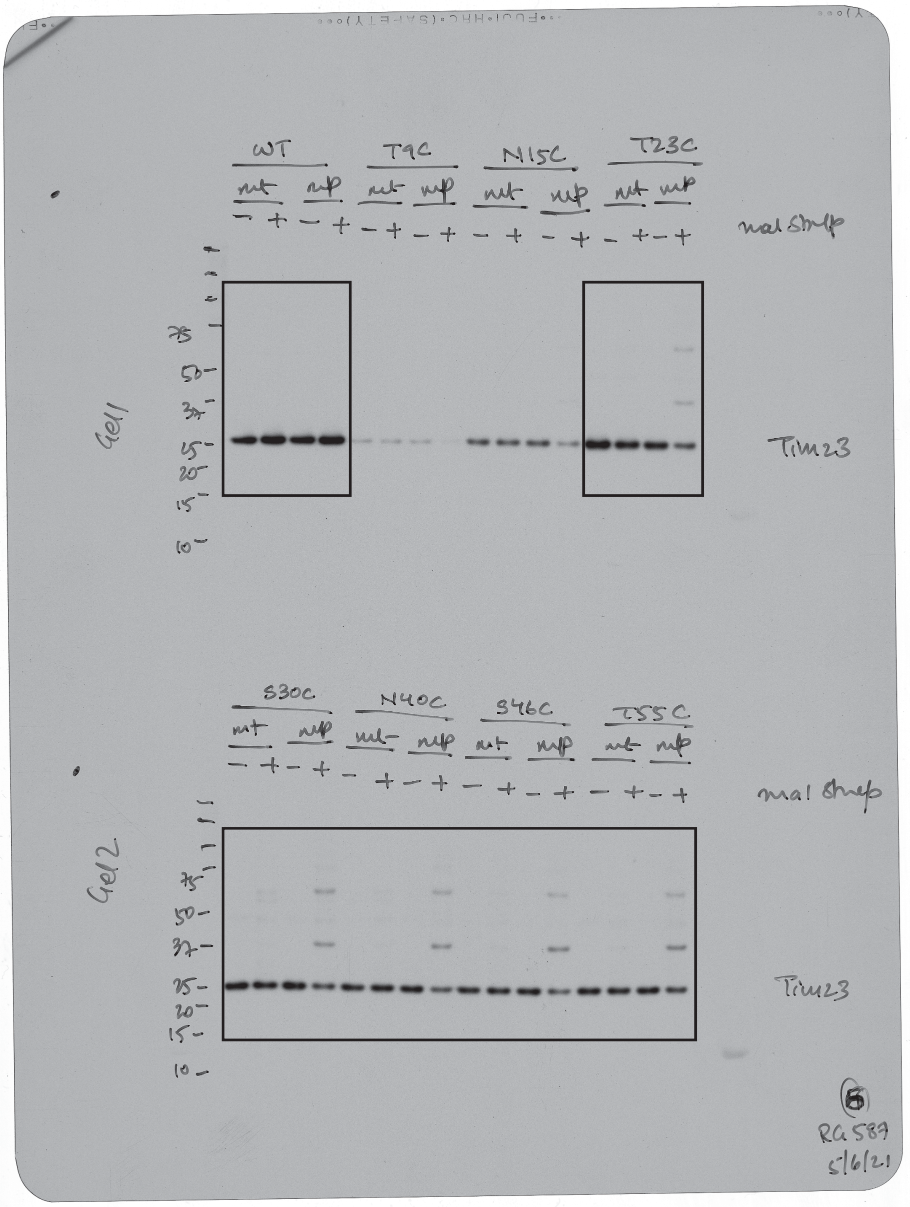

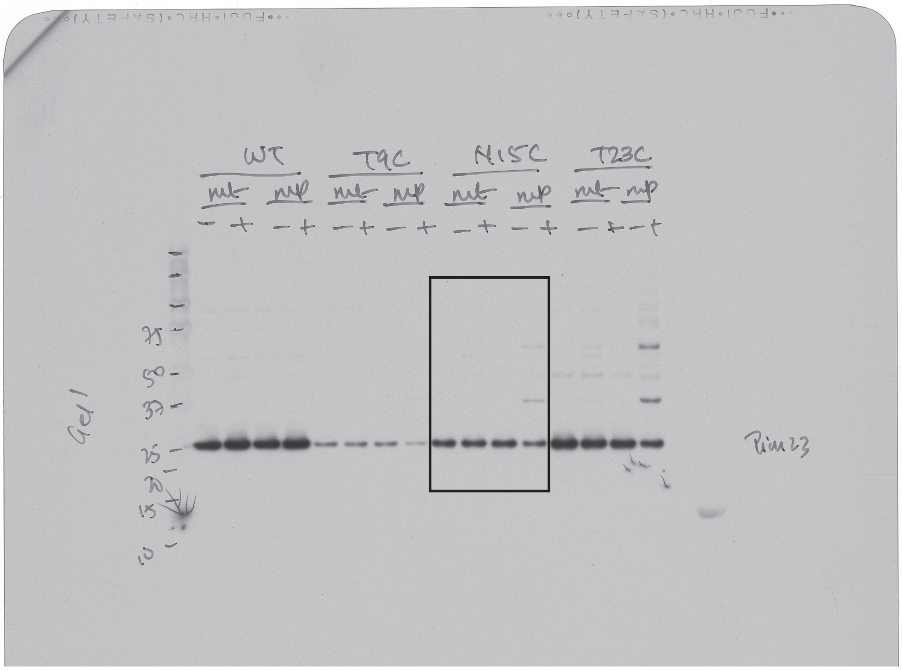

Supplement: Supplementary file 7 — Source Data [file 41467_2021_26016_MOESM7_ESM.zip › Source Data-gel images.pdf]
